# Global Coverage of Mandatory Large-Scale Food Fortification Programs: A Systematic Review and Meta-Analysis

**DOI:** 10.1016/j.advnut.2023.07.004

**Published:** 2023-07-25

**Authors:** Fabian Rohner, James P. Wirth, Wu Zeng, Nicolai Petry, William E.S. Donkor, Lynnette M. Neufeld, Penjani Mkambula, Sydney Groll, Mduduzi NN. Mbuya, Valerie M. Friesen

**Affiliations:** 1 GroundWork, Fläsch, Switzerland; 2 Georgetown University, School of Health, Washington DC, United States; 3 Food and Agriculture Organization of the United Nations (FAO; formerly GAIN); 4 Global Alliance for Improved Nutrition (GAIN), Geneva, Switzerland

**Keywords:** fortification, coverage, salt, maize, wheat flour, sugar, rice, vegetable oil

## Abstract

Food fortification with micronutrients is widely implemented to reduce micronutrient deficiencies and related outcomes. Although many factors affect the success of fortification programs, high population coverage is needed to have a public health impact. We aimed to provide recent global coverage estimates of salt, wheat flour, vegetable oil, maize flour, rice, and sugar among countries with mandatory fortification legislation. The indicators were the proportion of households consuming the: food, fortifiable food (that is, industrially processed), fortified food (to any extent), and adequately fortified food (according to national or international standards). We estimated the number of individuals reached with fortified foods. We systematically retrieved and reviewed all applicable evidence from: published reports and articles from January 2010 to August 2021, survey lists/databases from key organizations, and reports/literature received from key informants. We analyzed data with R statistical package using random-effects meta-analysis models. An estimated 94.4% of households consumed salt, 78.4% consumed fortified salt (4.2 billion people), and 48.6% consumed adequately fortified salt in 64, 84, and 31 countries, respectively. Additionally, 77.4% of households consumed wheat flour, 61.6% consumed fortifiable wheat flour, and 47.1% consumed fortified wheat flour (66.2 million people) in 15, 8, and 10 countries, respectively, and 87.0% consumed vegetable oil, 86.7% consumed fortifiable oil, and 40.1% consumed fortified oil (123.9 million people) in 10, 7, and 5 countries, respectively. Data on adequately fortified wheat flour and vegetable oil and coverage indicators for maize flour, rice, and sugar were limited. There are major data gaps on fortification coverage for most foods except salt. All countries with mandatory fortification programs should generate and use more coverage data to assess program performance and adjust programs as needed to realize their potential to reduce micronutrient deficiencies (PROSPERO CRD42021269364).

**Statement of Significance**

This is the first systematic review estimating the global coverage of fortified food vehicles and the number of individuals reached by fortified foods. The review reveals an enormous data gap that will have to be closed to identify the strengths and weaknesses of fortification programs and successfully address micronutrient deficiencies.

## Introduction

Globally, 1 in 2 preschool-aged children and 2 in 3 women of reproductive age are affected by ≥1 micronutrient deficiency [1]. Micronutrient deficiencies can have considerable negative consequences on an individual’s survival and mental and physical development, which in turn negatively affects the economies of countries as a whole [2,3]. Micronutrient deficiencies can be caused by low micronutrient intake due to a lack of dietary diversity, suboptimal absorption, and/or increased micronutrient losses due to infections [4].

Food fortification, or the addition of micronutrients lacking in a population’s diet to commonly consumed foods at the point of processing, is a widely used and sustainable approach to increasing micronutrient intakes at a large scale [5]. It has been shown to reduce micronutrient deficiencies and improve functional outcomes, such as reduced odds of developing anemia, goiter, or neural tube defects [6]. Food fortification first began in the early 1900s with the parboiling of rice in the Philippines [7]. This was followed in the 1920s with the addition of iodine to salt in Switzerland and the United States [8]. Subsequently, other micronutrients were added to cereals and milk in the United States and Canada [9,10]. It then expanded to sugar in Latin America in the 1970s, salt globally in the 1990s [11], and other staple foods and condiments in Africa and Asia in the 2000s [12].

Although many factors affect the success of food fortification programs [13], high population coverage is needed to have a public health impact. To understand the extent to which fortified foods are reaching a meaningful proportion of the population and in amounts that are sufficient to shift the distribution of micronutrient intakes toward adequacy, it is ideal to track changes in population-level coverage and consumption patterns of fortified foods at regular intervals throughout the implementation of a fortification program [13]. Such assessments should collect information on key indicators that provide evidence of delivery effectiveness and identify potential programmatic bottlenecks. Different measures of coverage specific to food fortification programs that serve this purpose have been previously defined and collected in several countries [14,15].

Despite the advantages of tracking coverage, this information is not routinely collected for most fortified foods [16]. Globally, Demographic Health Surveys (DHS) and UNICEF’s Multiple Indicator Cluster Surveys (MICS) regularly collect information on the household coverage of fortified salt but not of other commonly-fortified staple foods [17,18]. Other surveys that assess coverage of multiple fortified foods at national- or subnational levels, such as Fortification Assessment Coverage Toolkit (FACT) surveys [19], have been conducted sporadically in several countries [14,15]. Although some efforts have been made to consolidate the findings from FACT surveys across countries to understand common programmatic bottlenecks [16], a comprehensive assessment of all available coverage data has not yet been conducted to our knowledge.

In this systematic review we provide coverage and population reach estimates of 6 widely fortified foods (that is, salt, wheat flour, vegetable oil, maize flour, rice, and sugar) in countries with mandatory food fortification legislation. To assess the performance of food fortification programs in different contexts, we aimed to estimate coverage and reach for the aforementioned foods by geographic region, country-level income classification, residence (that is, urban/rural), and subnational socioeconomic status. Finally, we aimed to identify country-level factors that enable and limit the coverage of fortified foods.

## Methods

### Definition of outcome measures

We used different coverage indicators that are specific to food fortification programs as the outcome measures in this study [15]. These measures were previously defined by the Tanahashi model of health service coverage [14,20]. These indicators used are as follows:

1) proportion of households that consume a food (in any form),
2) proportion of households that consume a fortifiable food (that is, the food has been processed industrially or centrally and, therefore, has the potential to be fortified by a large-scale producer),
3) proportion of households that consume a fortified food vehicle (that is, the food is confirmed to be fortified to any extent), and
4) proportion of households that consume an *adequately fortified* food vehicle (that is, the food is confirmed to be fortified in accordance with national or international fortification standards).

The quantitative assessment of iodine for salt and vitamin A for vegetable oil and sugar was used to determine fortification adequacy. For foods that are fortified with >1 micronutrient using a micronutrient premix (for example, wheat flour, maize flour, rice), the fortification adequacy was typically assessed via the quantitative measurement of iron found in the food.

### Inclusion and exclusion criteria

This study included countries with mandatory food fortification legislation for ≥1 of the following foods: salt, wheat flour, vegetable oil, maize flour, rice, and sugar. Countries with voluntary food fortification legislation for these foods were excluded on the basis that they are more diverse in terms of delivery modalities (for example, only a few brands or only certain geographic areas) and are less likely to be systematically monitored.

The Population, Interventions, Comparators, and Study design (PICOS) criteria were used to define inclusion and exclusion of studies, although the “C” (Comparators) part of the PICOS approach did not apply, as this study does not compare 2 intervention groups. The PICOS description used for this study is presented in Table 1. In addition to the criteria presented below, any study for which data collection happened prior to 2010 was excluded.

**TABLE 1:** PICOS^1^ criteria for study selection

Parameter	Description
Population	Inclusion criteria: countries with mandatory food fortification legislation for ≥1 of the eligible foods; nationally representative surveys; or, if not nationally representative, covering ≥50% of the population.^2^ Exclusion criteria: convenience sampling yielding nonrepresentative data.
Interventions	Inclusion criteria: salt, wheat flour (including semolina), vegetable oil, maize flour, rice, and sugar. Exclusion criteria: any other food.
Comparators	Not applicable
Outcomes	Inclusion criteria: ≥1 of the 4 coverage indicators was assessed: proportion of households that consume a food, proportion of households that consume a fortifiable food, proportion of households that consume a fortified food, proportion of households that consume an adequately fortified food. Exclusion criteria: not reporting any of the coverage indicators.
Study design	Inclusion criteria: some level of population-representativeness, for example, used proportional to population size or random sampling.^2^ Exclusion criteria: studies using convenience or other non-random sampling, for example, using hospital volunteers, clinic or hospital patients; reviews, commentaries, letters to the editor, studies not conducted in humans.

Abbreviations: PICOS, Population, Interventions, Comparators, and Study design.

2 Although population-based national-level data were prioritized over data from regions within a country, we initially retained regional reports and, prior to extracting data, a country-specific decision was made on which data to extract. One notable exception relates to salt coverage where there were a high number of school-based studies; therefore, we considered school-based studies that used a sampling frame that yielded a representative sample.

### Search strategy

We performed a 3-pronged search strategy to identify relevant study reports or articles. This strategy was deemed necessary due to the assumed high proportion of survey reports that may not be published in the peer-reviewed literature. The search strategy is described in detail in the Supplementary Material 1 and presented briefly as follows.

First, we conducted a systematic search of studies published between 1 January, 2010 and 31 August, 2021 in relevant literature databases including PubMed, Scopus, Academic Search Premiere, and SciElo and a title search on Google Scholar. Detailed search terms are provided in Supplementary Material 1. No language restrictions were used.

Second, we reviewed global reporting databases that contained relevant documents including: Global Fortification Data Exchange (GFDx) [21], Iodine Global Network Scorecard [22], International Zinc Nutritional Consultancy Group list of national nutrition surveys assessing micronutrients (unpublished database), the WHO Vitamin and Mineral Nutrition Information System database [23], DHS Statcompiler (for fortified salt only) [24], MICS (for fortified salt only) [25], and International Household Survey Network [26].

Third, we contacted key informants made up of global and regional experts and stakeholders from organizations known to be active in the field of food fortification by email initially and, if necessary, by phone.

### Screening, study prioritization, and data extraction

Because reports and articles were obtained through multiple sources and formats, we used several Microsoft Excel files to capture the different data input formats and made daily data backups to avoid data losses. At the screening stage, we used 1 filing system to directly import titles and abstracts from the systematic literature search. Two reviewers (FR and SG) conducted a title screening followed by an abstract screening of retained titles using the PICOS criteria described above. Disagreements were resolved by direct discussion between the 2 reviewers. Reports obtained from global databases and experts and stakeholders were screened for eligibility and identifying information. Subsequently, all potentially eligible reports and articles were transferred to a separate database where source identifiers were inserted for each year from 2010 to 2021 (see Supplementary Material 1).

When multiple source documents were identified for a given country–food combination, the most recent source document was reviewed and, if it contained extractable and complete data, the older source documents were excluded. Alternatively, if no extractable data were available in the most recent source document, the next most recent source document for that country–food combination was screened for extractable data. This process was repeated until extractable data could be identified or no more source documents were available.

Three reviewers (FR, WD, SG) extracted data from the identified source documents that were in English, and 1 reviewer (FR) extracted data from source documents in other languages. For source documents in English, French, Spanish, Portuguese, German, and Chinese, the data were extracted directly, whereas for documents in other languages, a translation software was used to determine whether reports contained extractable data. We extracted information on general study design, important contextual information (such as method of analysis to quantify the amount of added nutrient), analytical methods, the different coverage indicators for the entire study population and subgroups, such as residence (that is, urban or rural) and socioeconomic status (for example, wealth quintiles), if available. For coverage indicators, the sample size, point estimate, and precision estimates were extracted.

### Risk of bias assessment

Risk of bias was assessed using a tool developed by Hoy et al. [27] that assesses risk of bias for “prevalence studies.” The tool contains 11 criteria to assess risk of bias, and we modified criterion 7 (reliability and validity of instrument) to include the assessment of the analytical method used to quantify the micronutrients added to foods (Supplementary Material 1). No changes were made to the other criteria.

### Data synthesis and statistical analysis

We harmonized the outcomes of interest by converting the numerators and denominators to proportions where required, paying particular attention to the sampling approaches and associated sampling weights used in the studies. We pooled the country-specific proportions using random-effects meta-analysis, which correctly estimates variances even in the presence of extreme proportions close to 0 or 1 and constrains CIs to within the 0–1 range after back transformation [28,29]. We calculated funnel graphs and statistics, such as Tau^2^ and I^2^, to assess the heterogeneity of the pooled coverage. We assessed between-study heterogeneity to judge the feasibility of subgroup meta-analysis by region, country income category, residence, and socioeconomic status. To generate summary coverage estimates by population group, we applied a random-effects model to perform the meta-analysis.

Where sufficient data were available, the fortified food coverage estimates for each country were multiplied by population estimates from the World Bank [30] to generate country-level population reach estimates. Population estimates were matched to the year of data collection of each specific data source. For example, if a country had data on the coverage of fortified wheat flour collected in 2014, the country’s population estimate from 2014 was used when calculating the number of individuals covered with that fortified food. We aggregated these country-level estimates to estimate the population reach in total and by subgroups for the food. For countries with mandatory food fortification legislation but no recent coverage data, population data from the median year (that is, 2017) of all included coverage data were used when summing the population of countries with survey data. In cases when coverage data were available from 2021, the 2020 population figures were used. Where sufficient data were available, we estimated population reach by geographic region and country-level income group using the World Bank classifications [31]. As some of the World Bank regions had very few data points, we merged similar regions into larger regions as follows: Americas = “North America” + “Latin America and the Caribbean”; South Asia, East Asia, and Pacific = “South Asia” + “East Asia & Pacific”; other regions are as per the World Bank definition.

Prior to searching for coverage data for the various foods, we aimed to use a meta-regression to examine the factors that contributed to the coverage of the fortified food. Following the completion of the data search, sufficient coverage data to permit a meta-analysis were only available for salt iodization programs. Using a framework developed as part of the study protocol, we examined the numerous national-level indicators that could affect the fortification coverage. These national-level indicators were matched, when possible, to the year the coverage data were collected. For the meta-regression, the dependent variable was the proportion of households consuming the fortified salt. The independent variables included measures of country-level socioeconomic status, governance, infrastructure, and food systems. Independent variables were drawn from the Human Development Report [32], the Food Systems Dashboard [33], the World Bank [34], and the GFDx [35]. Further details about the specific variables explored are provided in Supplementary Material 2. Prior to building the model, we calculated the variance inflation factor for all pairs of independent variables and used variance inflation factor >10 to identify collinearity. When cases of collinearity occurred, we determined which variables to retain and which to exclude based on data availability. Where there were sufficient fortified salt coverage data at the national level, by urban/rural areas, and for wealth quintiles, we developed 3 separate meta-regressions. In the meta-regression analysis for urban/rural areas, we dropped the independent variable on urbanization, as it was captured by the variable for urban or rural areas. All the analyses were performed using R Package 4.1.2 (R Foundation).

### Ethical considerations

This study protocol was granted exemption from a full review by the Institutional Review Board of the Georgetown University, USA (STUDY00004128). The study protocol was registered on PROSPERO (CRD42021269364).

## Results

### Search results and sources of information

**FIGURE 1:**
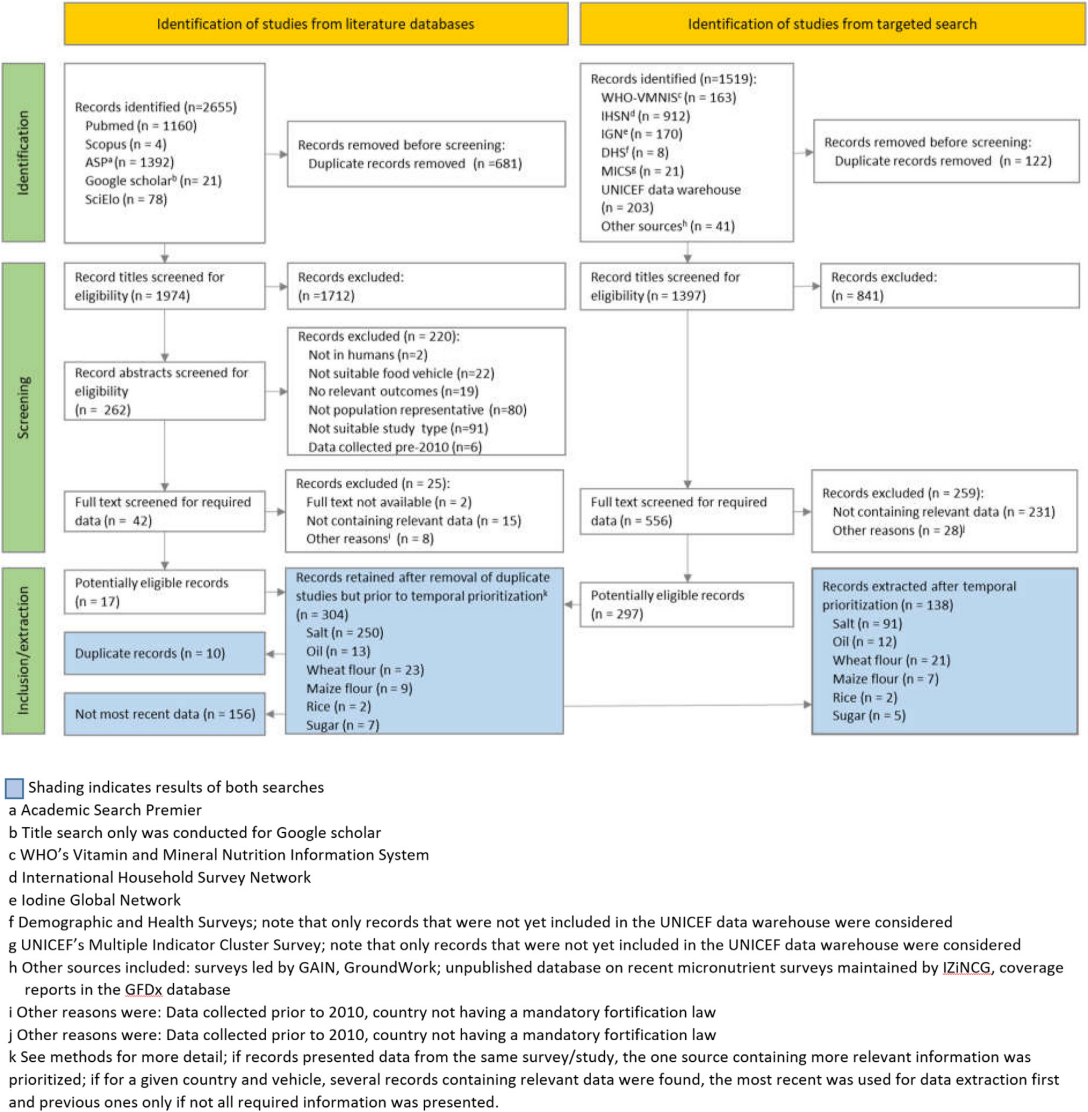
PRISMA flowchart of the literature search for selection and inclusion of articles and reports. ASP; DHS, Demographic and Health Survey; GAIN, Global Alliance for Improved Nutrition; GFDx, Global Fortification Data Exchange; IGN, Iodine Global Network; iZiNCG, international Zinc Nutritional Consultancy Group; MICS, Multiple Indicator Cluster Survey; VMNIS, Vitamin and Mineral Nutrition Information System.

We identified a total of 4174 records including 2655 from peer-reviewed literature databases and 1519 from the targeted search of institutional websites and databases and reports/articles shared by key experts (Figure 1). Responses were received from 20 international nutrition experts working for UN agencies, donor agencies, academia, international nongovernmental organizations and the private sector. The peer-reviewed literature search yielded 17 articles that were potentially relevant for this review, and the targeted literature search yielded 297 full texts. After merging the records from the search strategies, 10 duplicate records were removed, yielding 304 records for the various foods (blue central box; Figure 1).

Only the most recent record for each country–food combination was extracted (as described in the methods) except in 4 instances where 2 data sources provided complementary information from surveys conducted within 1.5 y (that is, all on salt coverage: Ethiopia, Gambia, Ghana, Malawi).

### Geographical and temporal distribution of coverage data

**FIGURE 2:**
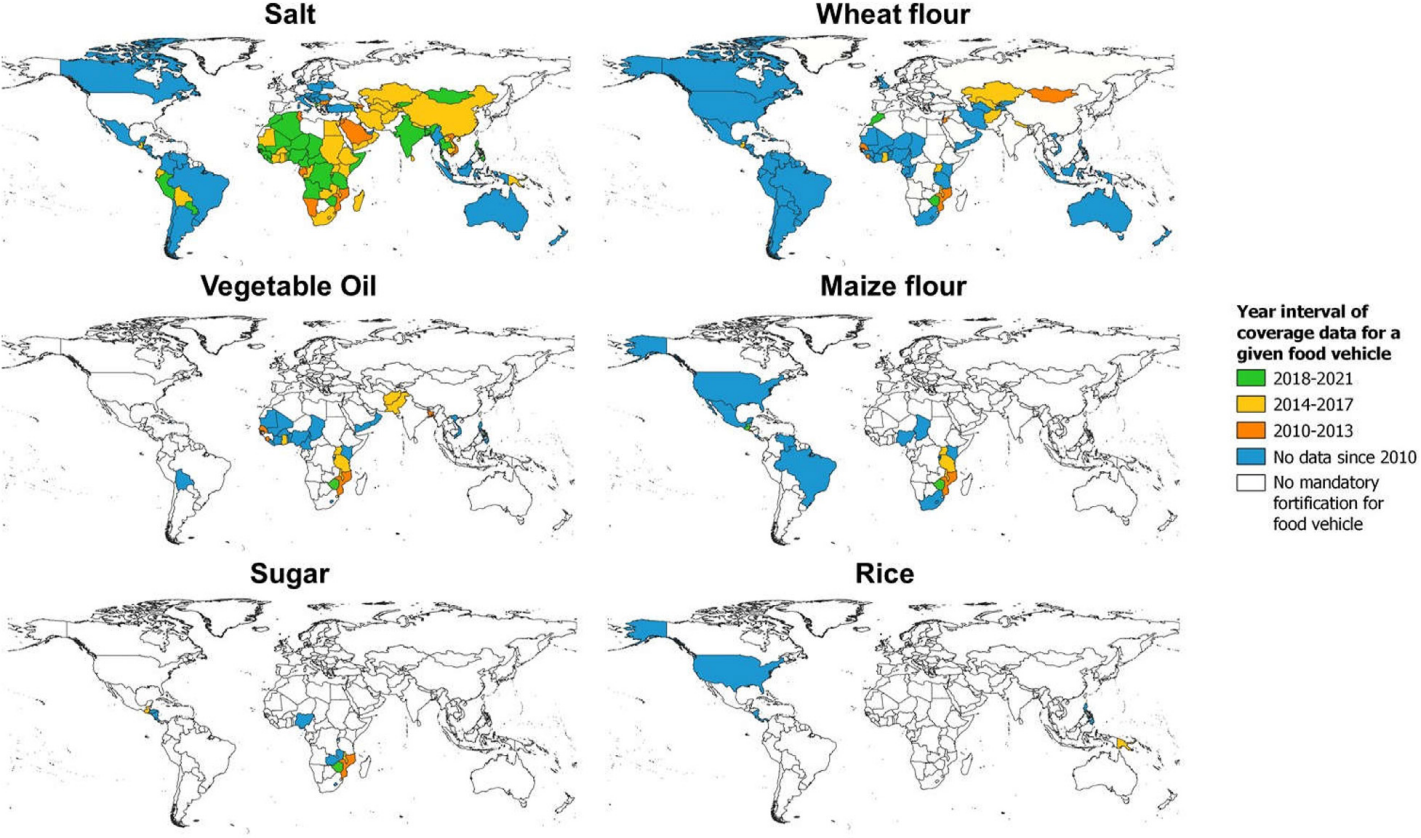
The availability of coverage data collected between 2010 and 2021 among countries with mandatory food fortification legislation as of June 2021. Number of countries with mandatory food fortification legislation included: salt, *n* = 125; wheat flour, *n* = 91; vegetable oil, *n* = 32; maize flour, *n* = 19; sugar, *n* = 12, and rice, *n* = 7. Coverage data included any of the following indicators: the proportion of households consuming the: *1*) food (in any form), *2*) fortifiable food (that is, industrially or centrally processed), *3*) fortified food (that is, fortified to any extent), and *4*) adequately fortified food (that is, fortified in accordance with national or international fortification standards).

Among countries with mandatory food fortification legislation as of June 2021, the availability of coverage data collected between 2010 and 2021 varied greatly by food and country (Figure 2). For salt, nearly two-thirds of all countries in the world have mandatory salt fortification legislation (*n* = 125), and nearly all countries on the African continent and in Central and South Asia have recent coverage data. In contrast, there were either no coverage data or data from before 2010 for many countries in Europe and the Americas. For wheat flour, almost half of all countries in the world (*n* = 91), mostly in the Americas, West Africa, and East Africa, have mandatory fortification legislation; however, only 20% of these countries have collected coverage data since 2010. For vegetable oil, only 32 countries have mandatory fortification programs and are mostly found in West and East Africa, whereas some are in South America and Asia. However, less than half of the countries with mandatory vegetable oil fortification legislation have collected coverage data since 2010. For maize flour, only 19 countries in the Americas and Africa have mandatory fortification legislation and only about one-quarter of them have collected coverage data since 2010. Finally, for sugar and rice, only 12 and 7 countries have mandatory fortification legislation, respectively, and coverage data since 2010 was only available in 5 countries for sugar and 2 countries for rice. Detailed results on coverage data by food and country, including the exact year of data collection, are provided in Supplementary Material 3.

### Global coverage estimates

#### Salt

Out of the 125 countries with mandatory food fortification legislation, an estimated 94% of households consume salt (in any form), 96% consume fortifiable salt, 78% consume fortified salt, and 49% consume adequately fortified salt based on coverage data from 64, 3, 84, and 31 countries, respectively (Table 2). For coverage of salt (in any form) and fortifiable salt, similar trends were observed by region, income group, residence, and socioeconomic status subgroups with coverage >89% in all subgroups. For fortified salt and adequately fortified salt, coverage tended to be higher among households with higher wealth (that is, income group and socioeconomic status) and urban residence compared with those with lower wealth and rural residence. Additionally, for adequately fortified salt, some differences were observed by region with the lowest coverage being in the Americas (30%) compared with the highest being in Europe and Central Asia (85%).

**TABLE 2:** Coverage estimates for salt among countries with mandatory food fortification legislation by subgroup

Group	Subgroup	N_countries mandatory_^2^	Coverage indicator^1^							
			Household consumes the food		Household consumes the fortifiable food		Household consumes the fortified food		Household consumes the adequately fortified food	
			N_countries with data_^3^	% (95% CI)	N_countries with data_^3^	% (95% CI)	N_countries with data_^3^	% (95% CI)	N_countries with data_^3^	% (95% CI)
Total		125	64	94.4 (93.2, 95.6)	3	96.3 (89.2, 100.0)^4^	84	78.4 (73.9, 83.0)	31	48.6 (39.2, 57.9)
Region^5^	Sub-Saharan Africa	44	37	93.1 (92.0, 94.3)	0	—6	41	74.6 (67.7, 81.4)	11	30.2 (21.1, 39.2)
	Americas	21	3	94.1 (92.2, 96.0)	0	—6	7	75.1 (51.3, 98.9)	5	64.6 (42.2, 87.1)
	South Asia, East Asia, and Pacific	35	12	93.9 (89.5, 98.4)	1	100.0 (100.0, 100.0)	15	80.5 (71.2, 89.9)	3	57.9 (35.4, 80.4)
	Europe and Central Asia	18	8	99.4 (99.0, 99.8)	1	99.0 (99.8, 100.0)^4^	10	85.0 (73.8, 96.1)	4	72.6 (48.8, 96.4)
	Middle East and North Africa	6	4	98.1 (95.4, 100.0)^4^	1	89.0 (87.9, 90.1)	11	86.1 (78.3, 93.9)	8	48.2 (28.0, 68.4)
Income	Low	26	21	93.1 (91.1, 95.1)	1	100.0 (100.0, 100.0)	23	67.7 (57.4, 78.0)	8	22.9(11.4, 34.4)
	Lower middle	39	33	94.6 (92.9, 96.4)	1	89.0 (87.9, 90.1)	40	80.6 (74.4, 86.8)	14	48.6 (38.1, 589.0)
	Upper middle	38	10	96.5 (94.2, 98.8)	1	99.0 (99.8, 100.0)^4^	18	88.4 (82.3, 94.5)	6	76.4 (57.6, 95.3)
	High	21	0	—	0	—	3	71.2 (54.4, 88.0)	3	60.5 (37.3, 83.7)
Residence	Urban	124	57	94.7 (93.5, 95.8)	3	97.7 (93.4, 100.0)^4^	60	80.5 (75.3, 85.7)	13	43.9 (26.0, 61.8)
	Rural	124	57	94.7 (93.3, 96.1)	3	93.3 (80.2, 100.0)^4^	59	75.6 (70.1, 81.1)	12	33.1 (18.9, 47.2)
Socioeconomic status^7^	Poorest	124	53	91.8 (89.5, 94.1)	0	—6	54	72.0 (66.3, 77.7)	8	31.1 (16.5, 45.6)
	Second	124	53	93.5 (91.9, 95.1)	0	—6	54	75.6 (69.9, 81.3)	8	35.9 (20.8, 51.0)
	Middle	124	53	94.1 (92.7, 95.5)	0	—6	54	78.3 (72.9, 83.8)	8	35.8 (18.9, 52.8)
	Fourth	124	53	94.7 (93.5, 95.9)	0	—6	54	80.5 (75.0, 85.9)	8	39.3 (21.1, 57.5)
	Wealthiest	124	53	96.7 (95.9, 97.4)	0	—6	54	82.9 (77.2, 88.7)	8	43.8 (23.5, 64.1)

1 Coverage indicators were defined as the proportion of households consuming the: *1*) food (in any form), *2*) fortifiable food (that is, industrially or centrally processed), *3*) fortified food (that is, fortified to any extent), and *4*) adequately fortified food (that is, fortified in accordance with national or international fortification standards).

2 Total number of countries with mandatory food fortification legislation as of June 2021 in total or by subgroup.

3 Total number of countries with mandatory food fortification legislation as of June 2021 and coverage data collected between 2010 and 2021.

4 The upper limit of the 95% CI was capped at 100%.

5 Region and income group were defined as per the World Bank Group classifications [31].

6 There were insufficient data available to conduct these subgroup analyses.

7 Socioeconomic status was defined as per country wealth quintiles.

Detailed country-level coverage results for salt and other foods are provided in tables in Supplementary Material 3 and as forest plots in Supplementary Material 4.

#### Wheat flour

Of the 91 countries with mandatory food fortification legislation, an estimated 77% of households consume wheat flour (in any form), 62% consume fortifiable wheat flour, 47% consume fortified wheat flour, and 35% consume adequately fortified wheat flour based on coverage data from 15, 8, 10, and 5 countries, respectively (Table 3). For all indicators, coverage tended to be higher among urban households compared with rural households.

**TABLE 3:** Coverage estimates for wheat flour, vegetable oil, maize flour, rice and sugar among countries with mandatory food fortification legislation by subgroup

Food/group	Subgroup	N_countries mandatory_^2^	Coverage indicator^1^							
			Household consumes the food		Household consumes the fortifiable food		Household consumes the fortified food		Household consumes the adequately fortified food	
			N_countries with data_^3^	% (95% CI)	N_countries with data_^3^	% (95% CI)	N_countries with data_^3^	% (95% CI)	N_countries with data_^3^	% (95% CI)
Wheat flour										
Total		91	15	77.4 (64.7, 90.1)	8	61.6 (41.6, 81.6)	10	47.1 (26.0, 68.2)	5	34.9 (27.8, 42.0)
Residence	Urban	91	10	67.4 (46.4, 88.4)	7	71.9 (53.5, 90.3)	6	49.3 (28.2, 70.5)	4	23.2 (12.9, 33.6)
	Rural	91	9^4^	57.7 (32.1, 83.3)	7	53.4 (29.6, 77.1)	6	35.7 (13.0, 58.4)	4	21.7 (5.9, 37.5)
Vegetable oil										
Total		32	10	87.0 (76.8, 97.1)	7	86.7 (71.7, 100.0)^5^	5	40.1 (28.9, 51.2)	2	33.7 (0.0, 76.5)^5^
Maize flourv										
Total		19	5	66.8 (36.7, 96.8)	2	38.7 (32.8, 44.6)	3	33.5 (0.0, 90.27)^5^	0	—
Rice										
Total		7	2	80.8 (51.8, 100.0)^5^	1	95.6 (95.0, 96.2)	1	95.6 (95.0, 96.2)	0	—
Sugar										
Total		12	2	68.6 (58.7, 78.6)	0	—	2	67.7 (12.0, 100.0)^5^	3	51.3 (19.7, 82.8)

1 Coverage indicators were defined as the proportion of households consuming the: *1*) food (in any form), *2*) fortifiable food (that is, industrially or centrally processed), *3*) fortified food (that is, fortified to any extent), and *4*) adequately fortified food (that is, fortified in accordance with national or international fortification standards).

2 Total number of countries with mandatory food fortification legislation as of June 2021 in total or by subgroup.

3 Total number of countries with mandatory food fortification legislation as of June 2021 and coverage data collected between 2010 and 2021.

4 For 1 country (Mozambique), only urban data were available.

5 The upper limit of the 95% CI was capped at 100%, and the lower limit was capped at 0.0%.

#### Vegetable oil

An estimated 87% of households consume vegetable oil (in any form), 87% consume fortifiable vegetable oil, 40% consume fortified vegetable oil, and 34% consume adequately fortified vegetable oil based on coverage data from 10, 7, 5, and 2 countries, respectively, out of the 32 countries with mandatory food fortification legislation (Table 3).

#### Maize flour

An estimated 67% of households consume maize flour (in any form), 39% consume fortifiable maize flour, and 34% consume fortified maize flour based on coverage data from 5, 2, and 3 countries, respectively, out of the 19 countries with mandatory food fortification legislation (Table 3).

#### Rice

Of the 7 countries with mandatory food fortification legislation, an estimated 81% of households consume rice (in any form), 96% consume fortifiable rice, and 96% consumed fortified rice based on coverage data from 2, 1, and 1 countries, respectively (Table 3).

#### Sugar

Of the 12 countries with mandatory food fortification legislation, an estimated 69% of households consume sugar (in any form), 68% consume fortified sugar, and 51% consume adequately fortified sugar based on coverage data from 2, 2, and 3 countries respectively (Table 3).

There were insufficient data available to estimate coverage of fortifiable sugar, coverage of adequately fortified maize flour and rice, and to conduct subgroup analyses for all foods except by residence for wheat flour. Where limited data were available, results should be interpreted with caution.

### Estimated population reached with fortified foods

Among countries with mandatory fortification, the number of individuals reached with fortified foods was estimated to be ∼4.2 billion for salt, 66.2 million for wheat flour, 123.9 million for vegetable oil, 9.6 million for maize flour, 0.5 million for rice, and 12.2 million for sugar (Table 4). Available data on fortified salt coverage represented almost 80% of people living in all countries with mandatory salt fortification legislation, but there were considerable differences by region and income group. By region, coverage data were available for all or most people (88%–100%) living in countries with mandatory salt fortification legislation in the Middle East and North Africa, Sub-Saharan Africa, and South Asia, East Asia, and the Pacific, but only a small proportion (15%–25%) of the population in the Americas and Europe and Central Asia. Similarly, by income group, coverage data were available for more than two-thirds of people living in low-income, lower-middle-income, and upper-middle-income countries with mandatory salt fortification legislation. In contrast, coverage data were available for only 10% of the population from high-income countries. The results on fortified salt coverage and population reached described in this study are likely representative for Sub-Saharan Africa, the Middle East and North Africa, and South Asia, East Asia, and the Pacific regions, as well as for low and lower- and upper-middle-income countries but not in other regions and high-income countries. For other foods, available data on fortified food coverage represented only a small proportion of people living in all countries with mandatory fortification legislation; 28% for vegetable oil and 0%–8% for wheat flour, maize flour, rice, and sugar. As such, the coverage and population reach estimates for these foods are likely underestimated and should be interpreted with caution.

**TABLE 4:** **Population reached with fortified salt, wheat flour, vegetable oil, maize flour, rice**, **and sugar in countries** with mandatory food fortification legislation

Region	Number of countries with mandatory food fortification legislation	Total population in countries with mandatory food fortification legislation^1^	Number of countries with data on fortified food coverage^2^	Population in countries with data on fortified food coverage^3^	Population reached with fortified food
Salt					
Total	125	6,070,948,602	84	4,787,181,263 (78.9%)	4,214,187,768
Region^4^					
Americas	21	649,607,683	7	100,420,258 (15.5%)	79,005,046
Europe and Central Asia	27	361,928,938	10	91,999,482 (25.4%)	72,019,518
Middle East and North Africa	15	356,182,944	11	331,793,008 (93.2%)	296,433,372
Sub-Saharan Africa	41	1,022,510,592	41	1,022,510,592 (100.0%)	825,252,391
South Asia, East Asia, and Pacific	21	3,680,718,445	15	3,240,457,923 (88.0%)	2,941,477,440
Income group^3^					
Low income	27	624,034,897	23	546,718,755 (87.6%)	388,897,614
Lower middle income	39	2,745,942,717	40	2,566,566,960 (93.5%)	2,255,654,801
Upper middle income	38	2,421,155,045	18	1,640,945,367 (67.8%)	1,546,693,054
High income	21	279,815,943	3	32,950,181 (11.8%)	22,942,299
Wheat flour					
Total	91	2,552,146,224	10	190,199,069 (7.5%)	66,247,286
Vegetable oil					
Total	32	1,229,936,282	5	347,692,837 (28.3%)	123,891,265
Maize flour					
Total	19	1,200,067,619	3	95,946,010 (8.0%)	9,555,870
Rice					
Total	7	454,745,961	1	571,329 (0.1%)	546,191
Sugar					
Total	12	322,040,251	2	22,065,612 (6.9%)	12,192,860

1 Population estimates were matched to the year of data collection of each specific data source. For countries with mandatory food fortification legislation but no recent coverage data, population data from 2017 were used, as this was the median year of all included coverage data. Population figures from 2010–2020 were obtained from the World Bank [30]. In cases when coverage data were available from 2021, the 2020 population figures were used.

2 Fortified food was defined as being fortified to any extent.

3 *N (%)*; Percentage of population in countries with data on fortified food coverage out of the total population in countries with mandatory food fortification legislation.

4 Region and income group were defined as per the World Bank Group classifications [31].

### Meta-regression to identify societal- and programmatic-level barriers and enablers of mandatory salt iodization programs

Good governance and the maturity of the fortification program were identified as country-level factors that enabled the coverage of fortified salt based on the results from 3 meta-regression models (Table 5). The governmental effectiveness index was positively and significantly associated with fortified salt coverage in both the national and income group models, but not the urban/rural model (see Supplementary Material 2 for more information on the index). The coefficients suggest that for every point increase in the index (which ranges from −2.5 to +2.5), the coverage of fortified salt increases by ∼15 percentage points.

**TABLE 5:** Fitted coefficients from mixed effects meta-regression of national-level factors associated with the household coverage of fortified salt

Variables	National model (*n* = 69)^1^		Urban or rural model (*n* = 96)^1^		Income group model (*n* = 230)^1^	
	Coefficient	(95% CI)	Coefficient	(95% CI)	Coefficient	(95% CI)
Urban	—	—	Reference		—	—
Rural	—	—	−4.08	(−11.18, 3.01)	—	—
Socioeconomic status 1 (poorest)	—	—	—	—	−11.07∗∗	(−18.67, −3.47)
Socioeconomic status 2	—	—	—	—	−7.43	(−15.03, 0.16)
Socioeconomic status 3 (middle)	—	—	—	—	−4.62	(−12.22, 2.98)
Socioeconomic status 4	—	—	—	—	−2.27	(−9.86, 5.33)
Socioeconomic status 5 (wealthiest)	—	—	—	—	Reference	
Year salt iodization was made mandatory	−0.19	(−0.61, 0.22)	−0.82∗∗	(−1.31, −0.39)	−0.97∗∗∗	(−1.31, −0.64)
Voice and Accountability Index	−2.38	(−10.62, 5.87)	−7.07	(−11.83, 3.33)	−5.13∗	(−10.13, −0.13)
Government Effectiveness Index	15.45∗	(0.19, 30.71)	14.29∗	(−1.55, 24.47)	15.40∗∗∗	(6.4, 24.39)
Percent of national population living in urban areas	−0.15	(−0.48, 0.18)	—	(−0.6, −0.04)	−0.17	(−0.37, 0.03)
Gini index	0.06	(−0.55, 0.67)	0.05	(−0.47, 0.49)	−0.04	(−0.36, 0.27)
Natural Log of gross domestic product per capita	5.49	(−3.54, 14.52)	−1.14	(−4.48, 11.59)	−1.02	(−7.14, 5.1)
Number of supermarkets per 100,000 population	−0.21	(−2.2, 1.77)	−0.53	(−2.86, 1.75)	−0.16	(−1.72, 1.4)
Ease of doing business index	0.04	(−0.12, 0.2)	−0.02	(−0.13, 0.13)	−0.01	(−0.1, 0.08)
Official development assistance per capita	0.01	(−0.05, 0.06)	0.04	(−0.06, 0.11)	0.03	(−0.03, 0.08)
Intercept	426.57	(−416.33, 1269.47)	1731.57∗∗∗	(831.35, 2695.75)	2048.84∗∗∗	(1372.77, 2724.92)
R^2^	22.9%		27.6%		30.2%	

∗, ∗∗, and ∗∗∗ represent *P* values <0.05, <0.01, and <0.001, respectively.

1 Three regressions were fitted using household coverage of fortified salt (%) for *1*) an entire country, *2*) urban and rural areas within each country, and *3*) household wealth quintiles (that is, socioeconomic status) within each country.

The year the mandatory salt fortification legislation was passed was also significantly associated with fortified salt coverage in the urban/rural and income group models. The association was negative, implying that 1 y earlier in passing mandatory salt fortification legislation was associated with ∼1% higher coverage of fortified salt. In the income group model, the households from the lowest socioeconomic status quintile had significantly lower (∼11%) fortified salt coverage compared with the household coverage of the highest wealth quintile (*P* < 0.01).

Finally, fortified salt coverage in rural areas tended to be lower than that in urban areas in the urban/rural model, but the difference was not statistically significant. Meta-regressions exploring factors influencing the coverage of other fortified foods were not developed due to the small number of data points available.

## Discussion

In this systematic review, we provided recent global coverage and population estimates for 6 widely fortified foods (that is, salt, wheat flour, vegetable oil, maize flour, rice, and sugar) among countries with mandatory food fortification legislation. We found that there are major gaps in data availability and quality of fortification coverage globally for most foods except salt. Based on the available data, the results revealed that a relatively high proportion of households consume the foods (in any form) (67%–94%) and that this proportion generally decreased as the coverage indicators became more specific (that is, moving from fortifiable food to fortified food, and from fortified food to adequately fortified food).

### Data availability issues

There were limited data available on the set of coverage indicators assessed in this study for most foods except salt. For salt, coverage data were widely available, which is unsurprising given their systematic inclusion in DHS and MICS surveys for the past 3 decades [36], which enabled an estimation of global fortified salt coverage in 2020 that was slightly higher than the estimate in this study (88% vs. 78%, respectively) [37]. For other foods, coverage data were much less available. Unlike salt, there are no recurring surveys that systematically assess the coverage of other fortified foods. Although some recent efforts have been made to estimate the coverage of multiple food fortification indicators more frequently and routinely, they have had mixed success. For example, household coverage indicators were successfully integrated into recurring Performance Monitoring and Accountability 2020 surveys in Kenya and Burkina Faso in 2018 [38,39], which was done to assess the feasibility of their integration in large recurring surveys, such as DHS or MICS. Following that, the indicators were further proposed for incorporation into the DHS [40]; however, they were only adopted for the optional nutrition module but not the core questionnaire. As such, it currently remains up to individual countries implementing fortification programs to decide whether and how to collect coverage data. Although the coverage of fortification programs can be included in countries’ program monitoring and evaluation activities, limited technical and financial resources and competing priorities are likely common barriers to collecting the needed data.

### Data quality issues

Of the available data, the main data quality issue was that most surveys assessed only some, but not all, of the 4 coverage indicators specific to food fortification, which limits the identification of programmatic bottlenecks and areas for improvement. For salt, coverage of fortifiable salt was the main indicator consistently missing in the available data. However, as nearly all salt is likely fortifiable (that is, not home-produced), the lack of assessing this indicator is less problematic than for other foods where home production is widespread (for example, maize and wheat flours in some contexts). In the latter case, understanding the coverage of the fortifiable maize or wheat flours is essential for understanding the potential for impact of fortification in a population. For other foods, the relatively recent adoption of the cascade of coverage indicators for fortification nascency may be another reason that partly explains why there were few countries that collected most or all the indicators during the time frame assessed in this study (2010–2021). For instance, FACT surveys were the first to propose this cascade of indicators, and the first survey results were published for Rajasthan, India in 2016 [41] and 8 countries in 2017 [14], and the detailed FACT manual and tools were only made publicly available in 2019 [19].

### The coverage cascade and its use for assessing program performance

Although previous studies that assessed the performance of fortification programs have been largely based on reviews of program documents and key informant interviews [[42], [43], [44], [45]], assessing the cascade of coverage indicators in sequential order enables a more straightforward assessment of fortification program performance by rapidly identifying programmatic bottlenecks because each level of coverage is dependent on the achievement of the previous one [14]. Despite the aforementioned data availability and data quality issues, we illustrate how this can be done using some results of this study.

For example, for vegetable oil, the results revealed that vegetable oil is a good choice of food for fortification as it is widely consumed in any form (87% of households) and in a fortifiable form (87% of households). As such, it has potential to reach a high proportion of the population if all the fortifiable oil is fortified. However, the coverage of fortified and adequately fortified vegetable oil is low (40% and 34%, respectively) indicating that the major bottlenecks are, first, that some processors are not fortifying at all and, second, that others are fortifying but not with the required amounts. In countries with mandatory vegetable oil fortification, program improvement efforts should thus focus on understanding and addressing the barriers to fortification in general for processors who are not yet fortifying. Subsequently, these efforts could also aim to improve fortification compliance among producers who are fortifying but not to standard.

Conversely, for maize flour, despite a high proportion of households consuming maize flour in any form (67%), the major bottleneck is that the coverage of fortifiable maize flour is relatively low (39%). This indicates a feasibility gap in the choice of maize flour as a food for fortification given that a substantial proportion of the population in countries with mandatory maize fortification do not consume industrially processed maize flour that is amenable to industrial fortification. As such, improving processors’ ability to comply with fortification standards would not result in an effective fortification program. Program improvement efforts should thus focus on understanding the fortifiable maize flour consumption patterns within the countries (for example, which population groups consume it and what their characteristics are, such as location, wealth status, etc.) to understand who could be reached with fortified maize flour and whether other fortified foods (with the same added micronutrients) or other micronutrient interventions would be more effective approaches to improving micronutrient intake.

The coverage cascade also shows that there is a similar decrease in the coverage of fortifiable salt and fortified salt (percentage point difference = 17.9%) as the coverage of fortifiable wheat flour to fortified wheat flour (percentage point difference = 14.5%). This is a surprising finding because the recurring cost of micronutrient premix has been previously identified as barrier to food fortification [46], yet the average cost of fortifying a metric ton salt is substantially lower than the price of fortifying a metric ton wheat flour [47,48]. If the cost of micronutrient premix affected the coverage of fortified foods, we would have expected to find a smaller decrease in the coverage estimates of fortifiable and fortified salt.

### Enabling factors associated with fortified salt coverage

The meta-regression results revealed that good governance and the maturity of the program (that is, the number of years since the mandatory legislation was passed) were key factors that positively influenced the coverage of fortified salt. Other researchers have qualitatively identified effective governmental institutions as being critical for sustaining salt iodization programs [13,49], and our study demonstrates this association quantitatively. To assess good governance, we used the World Bank’s Governmental Effectiveness Index, which is a composite index based on the quality of a government’s public services, civil service, policy formulation, and policy implementation [50]. Although the use of this index as an independent variable does not permit the identification of specific governmental factors that are linked to improved program performance, governmental bodies are frequently responsible for food fortification compliance [13], and higher levels of governmental effectiveness could result in increased funding and/or technical capacity to support fortification program implementation and monitoring efforts. Poor enforcement by government regulators, limited laboratory capacity, and limited access to premix have been previously cited as barriers to effective fortification programs [51].

As salt fortification programs mature, the coverage of fortified salt increases. This finding is intuitive because the longer a program is implemented, the more likely it is that those involved (namely, governmental bodies and food processors) will become better at implementing their respective fortification-related tasks and will have identified and addressed any major issues limiting effective implementation. With more mature fortification programs, those responsible may also have had opportunities to adjust and adapt the type and frequency of program monitoring and evaluation activities and are more likely to have collected data on fortified food coverage.

### Strengths and limitations

The study had some notable strengths. First, we used a comprehensive search strategy that combined the conventional systematic search of peer-reviewed publications with reviews of global reporting databases and survey lists from key organizations and a targeted search among informants made up of global and regional experts, which increased the likelihood that all available literature and reports were identified. Second, we prioritized more recently collected data (when multiple data were available for a given country and it was of equivalent or higher quality than the older data), which resulted in the most recent snapshot of fortification coverage among countries with mandatory food fortification legislation. Third, we used meta-analysis techniques, which ensured that the results were objective and replicable and thus can facilitate a re-estimation of the global coverage of fortified foods to update these results in the future. Finally, the use of a meta-regression technique, which although only used for fortified salt enabled the quantitative identification of enablers and barriers of fortification programs.

The study also had some limitations. First, and most notably, there were limited coverage data available for most foods, except salt; therefore, the global coverage estimates may not be representative of all countries with mandatory food fortification legislation. Second, the study only included countries with mandatory food fortification legislation for ≥1 of the following foods: salt, wheat flour, vegetable oil, maize flour, rice, and sugar; therefore, the generalizability of the results to countries that voluntarily fortify these foods is limited. Future studies that examine the coverage of fortified foods in countries with voluntary fortification legislation are needed to estimate the coverage and potential impact of voluntary programs. Third, the study did not include other foods (for example, soy sauce or fish sauce [52]) that are mandatorily fortified in a small number of countries. Fourthly, our study did not have complete information about the funding and technical support provided to each food fortification program. Thus, we could not determine if external support from governments, foundations, or not-for-profit organizations was associated with a higher coverage of fortified foods.

## Conclusions

Food fortification programs have the potential to reduce micronutrient deficiencies and related outcomes when effectively designed to address documented inadequacy of nutrients in the population and implemented such that high population coverage of the fortified food is achieved. Our findings revealed that there are major gaps in fortification coverage data availability and quality for wheat flour, vegetable oil, maize flour, rice, and sugar, but not salt, among countries with mandatory food fortification legislation. The lack of coverage data in such contexts could lead to the unfounded assumptions that risk of deficiency is mitigated by such programs. We encourage all countries with mandatory fortification programs to generate and use the necessary coverage data to assess program performance and adjust programs as needed. Only with that evidence can the potential of mandatory fortification to reduce micronutrient deficiencies and related outcomes be realized.

## Acknowledgments

The Global Fortification Data Exchange data stewards helped to refine the scope of work and shared reports obtained through their activities: Florencia Vasta, Helena Pachón, Karen Codling, Becky Tsang, and Michelle Duong. When available, data on household coverage with fortified salt has been obtained from UNICEF’s global databases; where microdata was available, the Data and Analytics nutrition team re-analyzed the eligible datasets to match the need of this study. Monica Flores-Urrutia from WHO’s Vitamin and Mineral Nutrition Information System team provided potentially eligible reports the Iodine Global Network shared reports not obtained through a web-based search; we thank Maria Andersson for providing access to these resources. Several key stakeholders contacted as part of the search strategy responded to our enquiry with additional reports, and their efforts are acknowledged.

## Author contributions

The authors’ contributions were as follows—FR, JPW, WZ, LMN, PM, MNNM, VMF: designed research; FR, NP, WD, SG: conducted research; WZ, JPW: analyzed data; FR: wrote manuscript; WZ, JPW, LMN, PM, MNNM, VMF: critically reviewed manuscript; FR, VMF: have primary responsibility for final content; and all authors: read and approved the final manuscript.

## Conflict of interest

We declare that Global Alliance for Improved Nutrition is a not-for-profit organization supporting and promoting food fortification programs; PM, MNNM, and VMF are employees of Global Alliance for Improved Nutrition. The Bill & Melinda Gates Foundation as the funder had no role in study design, data collection and analysis, preparation of the manuscript, or decision to publish. All other authors have no conflicts of interest to declare. The views expressed in this publication are those of the authors and do not necessarily reflect the views of FAO.

## Funding

This work was supported, in whole or in part, by the Bill & Melinda Gates Foundation [INV-002077]. Under the grant conditions of the Foundation, a Creative Commons Attribution 4.0 Generic License has already been assigned to the Author Accepted Manuscript version that might arise from this submission.

## Data availability

All data used in this manuscript are presented in the manuscript or the supplementary material. Data described in the manuscript, code book, and analytic code will be made available upon request.

## Appendix A Supplementary data

Multimedia component1 Multimedia component1

Multimedia component2 Multimedia component2

Multimedia component3 Multimedia component3

Multimedia component4 Multimedia component4
