# Supplementary material for: Global Coverage of Mandatory Large-Scale Food Fortification Programs: A Systematic Review and Meta-Analysis
Source: Adv Nutr. 2023 Jul 25;14(5):1197–210. doi: 10.1016/j.advnut.2023.07.004 (PMC10509437; doi:10.1016/j.advnut.2023.07.004)
Supplement: Multimedia component1 [file mmc1.docx]

# Literature search

This systematic review and meta-analysis includes both published and unpublished reports and articles that may contain data relating to one or several outcomes of interest. The project aimed at comprehensively identifying such reports/articles in order to extract data as reported, but did not aim at obtaining individual participant data or other types of data containing participant identifiers. In case that there were reports indicating that information on the outcomes of interest was collected in a survey or study but not reported, we attempted to contact the authors of such a report or article and asked if they were willing to calculate the outcome of interest in question and share the results only. Authors were requested to share the actual underlying microdata nor any information containing participants identifiers.

This systematic review included a review of iodized salt coverage indicators collected as part of Demographic and Health Surveys (DHS) and Multiple Indicator Cluster Surveys (MICS). Both DHS and MICS do provide de-identified open-access microdata for completed surveys. However, to this extent, the systematic review consisted primarily of reviews of country-level databases (e.g. DHS Statcompiler) and reviews of DHS and MICS surveys reports. When available, data on household coverage with iodized salt has been obtained from UNICEF’s global databases (1) and upon the authors’ request and where microdata was available, the UNICEF Data and Analytics nutrition team re-analyzed the eligible datasets to match the need of this study. This re-analysis was done to obtain coverage estimates of the entire population and not only that segment with salt in the household at the time of the survey.

## Information sources and search strategy

As food fortification coverage results are frequently found in unpublished (but formally released) survey reports, multiple approaches were used to identify fortification coverage data. **Table 1** illustrates the search approach used for this project, and the below sections (1.1.1 - 1.1.3) provide more detail about each strategy of the search.

Table 1 Approach for searching and identifying data for systematic review and meta-analysis

| **Review approach** | **Source of articles and reports** | **Description of databases and websites** |
| --- | --- | --- |
| **1** | Review of micronutrient survey lists and databases to identify potentially suitable reports | - Global fortification data exchange (GFDx) (2) - Iodine Global Network Scorecard (3) - IZinCG national nutrition surveys assessing micronutrients list (unpublished database) - WHO VMNIS database (4) - DHS Statcompiler (for iodized salt only) (5) - MICS (for iodized salt only) (6) - International Household Survey Network – IHSN (for coverage of food vehicle) (7) |
| **2** | Consultation with experts and stakeholders from the following organizations or initiatives will be contacted | - Global Fortification Data Exchange stakeholders - The Global Alliance for Improved Nutrition (GAIN) - GroundWork - International Micronutrient Malnutrition Prevention and Control group, US CDC - Institut de Recherche pour le Développement (IRD), France - UNICEF regional nutrition representatives - World Food Programme (WFP) regional nutrition representatives - Nutrition International, Canada - Helen Keller International, US - International Food Policy Research Institute, US - World Health Organization - USAID - BMGF - Food Fortification Advisory Service (2FAS) - International Zinc Nutrition Consultative Group (IZinCG) |
| **3** | County-specific systematic review of peer-reviewed publications | - Google Scholar ^a^ - PubMed - Scopus - Academic search premier - SciElo (Spanish) |

^a^ Due to the different search algorithm compared to other literature databases, a title search in Google Scholar was used to yield a manageable number of references.

### Approach I – Review of existing fortification-related databases

As previously mentioned, a multi-pronged approach was used to conduct as separate systematic review for each food vehicle. The first approach consisted of a thorough review of fortification-related databases to identify population-based surveys and studies that have examined the coverage of fortified foods and food vehicles in general. Using the GFDx database (8) as a starting point and using additional decrees and other legal documents, countries that have mandatory or voluntary fortification of the relevant food vehicles were identified. The GFDx was set as a starting point since it is updated on an annual basis by multiple institutions (e.g., GAIN, FFI, IGN), as is currently the most comprehensive database on food fortification for all food vehicles in this study, except for sugar. The database contains references to surveys and studies that assessed the coverage of food fortification programs, and where available, presents summary information on the coverage of various fortified foods.

The GFDx database was complemented by reviews of other databases with information about the coverage of various fortified foods. Three database that contain results on the coverage of iodized salt include the Iodine Global Network Scorecard (3), DHS Statcompiler (5), and UNICEF’s MICS survey database (6). Other databases that contain multiple food vehicles, such as IZinCG’s National Micronutrient Surveys list (unpublished database), and the WHO VMNIS database (4) were also reviewed. The Central Data Catalogue of the International Household Survey Network (IHSN) (9) was also be used to search the key variables collected by household surveys. The IHSN is a comprehensive database of household consumption and expenditure surveys that frequently contain a 14-day daily household consumption and expenditure diary.

All potentially relevant source documents collected from each database were downloaded. If multiple surveys/studies were identified for the same country and food vehicle, the specific indicators/data contained in each survey/study was explored to determine its utility. In general, the most current data will for each country/food vehicle/indicator was used, however, the research team prioritized a) nationally-representative data over subnational-representative data, and b) quantitative data over qualitative data.

### Approach II – Consultation with experts

To identify other survey reports that may not have been published or added to the lists described in Section 1.1.1, the research team consulted with global or regional experts and stakeholders of food fortification programs. The agencies that were contacted are presented in **Table 1**.

The research team contacted fortification experts/stakeholders working at the global or regional levels; the research team deemed contacting national offices of all organizations infeasible. Individuals were contacted by email and asked to note the population-based surveys that have examined the coverage of the relevant fortified foods. The food specific datafiles were shared, as relevant, with the stakeholders so that they saw the data already compiled to determine if the surveys they were aware of would increase the data already collected as part of the project. In addition, the research team highlighted notable data gaps when communicating with the fortification experts/stakeholders with the aim of identifying new surveys.

### Approach III – Country-specific review of peer-reviewed literature

The systematic review of literature databases was conducted to identify any additional relevant survey reports or articles that were missed during phase 1 and phase 2.

Keyword searches were conducted in multiple literature databases, including PubMed, Scopus, Academic Search Premier, Google Scholar, and SciElo. A search in Spanish and French was considered necessary for this project as many countries in Central and South America and Francophone Africa have mandatory food fortification programs and since there are numerous peer-reviewed journals published only in Spanish and a few in French.

Search *terms*

Table 2 presents the search terms used to search selected literature databases. The search terms were developed to yield a comprehensive and relevant set of articles for each country- and food-specific search. The searches in the aforementioned databases search articles’ titles, abstracts, and key words.

Test searches in Google Scholar — which scans the full text of articles by default — yielded an unwieldy number of articles (>20,000). Google Scholar searches can be restricted to scan titles only, but the comprehensive search strings used to identify articles in the other databased cannot be used for Google Scholar title searches. Thus, separate search terms were used for a Google Scholar title search (see **Table 2**).

After compiling the list of article titles from the various literature databases, duplicates were identified and removed, resulting in a consolidated list of articles.

Table 2 Search terms for systematic search

| **Food vehicle** | **Search terms for Pubmed, Scopus, ASP, Scielo, Cochrane** | **Search terms for Google Scholar (Title search only)** |
| --- | --- | --- |
| Salt | (Afghanistan OR Albania OR Algeria OR Angola OR Argentina OR Armenia OR Australia OR Austria OR Azerbaijan OR Bahrain OR Bangladesh OR Belarus OR Belize OR Benin OR Bolivia OR Bosnia OR Brazil OR Bulgaria OR Burkina Faso OR Burundi OR Cambodia OR Cameroon OR Canada  “Cape Verde” OR “Cabo Verde” OR “Central African Republic” OR Chad OR Chile OR China OR Colombia OR Congo OR Congo-DRC OR “Costa Rica” OR “Côte d'Ivoire” OR “Ivory Coast” OR Croatia OR Denmark OR “Dominican Republic” OR Ecuador OR Egypt OR “El Salvador” OR Eswatini OR Ethiopia  Fiji OR Gabon OR Gambia OR Georgia OR Ghana OR Guatemala OR Guinea  Guinea-Bissau OR Haiti OR Honduras OR Hungary OR India OR Indonesia OR Iran OR Italy OR Jordan OR Kazakhstan OR Kenya OR Kiribati OR Kosovo OR Kuwait OR Kyrgyzstan OR “Lao PDR” OR Lebanon OR Lesotho OR Liberia OR Lithuania OR Macedonia OR Madagascar OR Malawi OR Malaysia OR Mali OR Mauritania OR Mexico OR Moldova OR Mongolia OR Morocco OR Mozambique OR Myanmar OR Namibia OR Nepal OR “New Zealand” OR Nicaragua OR Niger OR Nigeria OR Oman OR Palestine OR Panama OR “Papua New Guinea” OR Paraguay OR Peru OR Philippines OR Poland OR Qatar OR Romania OR Rwanda OR “Sao Tome and Principe” OR “Saudi Arabia” OR Senegal OR Serbia OR “Sierra Leone” OR Slovakia OR Slovenia OR “Solomon Islands” OR Somalia OR “South Africa” OR “Sri Lanka” OR Sudan OR Tajikistan OR Tanzania OR Thailand OR Togo OR Tunisia OR Turkey OR Turkmenistan OR Uganda OR “United Arab Emirates” OR Uruguay OR Uzbekistan OR Venezuela OR “Viet Nam” OR “Vietnam” OR Yemen OR Zambia OR Zimbabwe) AND (“salt” OR “NaCl”) AND (“iodized” OR “iodised” OR “iodine” OR “iodate” OR “iodated” OR “iodide” OR “fortification” OR “fortified” OR “enriched” OR “micronutrient”) AND (“coverage” OR “survey” OR “study” OR “assessment” OR “evaluation”) AND (“household” OR “woman” OR “women” OR “children” OR “child” OR “population” OR “intake” OR “consumption”) NOT (review) | 1. Salt AND iodine AND coverage 2. Salt AND iodization AND coverage |
| Wheat flour | (Afghanistan OR Antigua OR Argentina OR Australia OR Bahamas OR Bahrain OR Barbados OR Belize OR Benin OR Bolivia OR Brazil OR Burkina Faso OR Burundi OR Cameroon OR Canada OR Cape Verde OR Chad OR Tchad OR Chile OR Colombia OR Congo OR Costa Rica OR Côte d'Ivoire OR Ivory Coast OR Cuba OR Djibouti OR Dominica OR Dominican Republic  Ecuador OR El Salvador OR Fiji OR Gabon OR Gambia OR Ghana OR Grenada OR Guatemala OR Guinea OR Guyana OR Haiti OR Honduras OR Indonesia OR Iran OR Jamaica OR Jordan OR Kazakhstan OR Kenya OR Kiribati OR Kosovo OR Kyrgyzstan OR Lesotho OR Liberia OR Malawi OR Mali OR Mauritania OR Mexico OR Moldova OR Mongolia OR Morocco OR Mozambique OR Nepal OR New Zealand OR Nicaragua OR Niger OR Nigeria OR Oman OR Palestine OR Panama OR Paraguay OR Peru OR Philippines OR Rwanda OR Saint Kitts OR Saint Lucia OR Saint Vincent OR Senegal OR Sierra Leone OR Solomon OR South Africa OR Suriname OR Tajikistan OR Tanzania OR Togo OR Trinidad OR Tobago OR Turkmenistan OR Uganda OR United Kingdom OR United States OR Uruguay OR Uzbekistan OR Venezuela OR Viet Nam OR Vietnam OR Yemen OR Zimbabwe) AND (“wheat” OR “semolina”) AND (“fortification” OR “fortified” OR “enriched” OR “micronutrient” OR “iron” OR “mineral” OR “vitamin”) AND (“coverage” OR “survey” OR “study” OR “assessment” OR “evaluation”) AND (“household” OR “woman” OR “women” OR “children” OR “child” OR “population” OR “intake” OR “consumption”) NOT (biofortification) NOT (review) | 1. Wheat AND fortification AND coverage 2. Wheat AND fortified AND coverage |
| Vegetable oil | (Afghanistan OR Bangladesh OR Benin OR Bolivia OR Burkina Faso OR Burundi OR Cameroon OR Chad OR Tchad OR Côte d'Ivoire OR Ivory Coast OR Djibouti OR Gambia OR Ghana OR Haiti OR Kenya OR Lesotho OR Liberia OR Malawi OR Mali OR Mauritania OR Mozambique OR Nigeria OR Oman OR Pakistan OR Philippines OR Senegal OR Sierra Leone OR Tanzania OR Togo OR Uganda OR Viet Nam OR Vietnam OR Yemen OR Zimbabwe) AND oil AND (“fortified” OR “enriched” OR “micronutrient” OR “retin*” OR “mineral” OR “vitamin”) AND (“coverage” OR “survey” OR “study” OR “assessment” OR “evaluation”) AND (“household” OR “woman” OR “women” OR “children” OR “child” OR “population” OR “intake” OR “consumption”) NOT (review) | 1. Oil AND fortification AND coverage 2. Oil AND fortified AND coverage |
| Maize meal | (Brazil OR Burundi OR Chad OR Tchad OR Costa Rica OR El Salvador OR Guatemala OR Kenya OR Lesotho OR Malawi OR Mexico OR Mozambique  Nigeria OR Rwanda OR South Africa OR Tanzania OR Uganda OR United States OR Venezuela OR Zimbabwe) AND (“maize” OR “corn”) AND (“fortified” OR “enriched” OR “micronutrient” OR “iron” OR “mineral” OR “vitamin”) AND (“coverage” OR “survey” OR “study” OR “assessment” OR “evaluation”) AND (“household” OR “woman” OR “women” OR “children” OR “child” OR “population” OR “intake” OR “consumption”) NOT (biofortification) NOT (review) | 1. Maize AND fortification AND coverage 2. Maize AND fortified AND coverage 3. Corn AND fortification AND coverage 4. Corn AND fortified AND coverage |
| Rice | (Costa Rica OR Nicaragua OR Panama OR Papua New Guinea OR Philippines OR Solomon Islands OR United States) AND (“rice”) AND (“fortification” OR “fortified” OR “enriched” OR “micronutrient” OR “iron” OR “mineral” OR “vitamin”) AND (“coverage” OR “survey” OR “study” OR “assessment” OR “evaluation”) AND (“household” OR “woman” OR “women” OR “children” OR “child” OR “population” OR “intake” OR “consumption”) NOT (biofortification) NOT (review) | 1. Rice AND fortification AND coverage 2. Rice AND fortified AND coverage |
| Sugar | (Costa Rica OR El Salvador OR Guatemala OR Honduras OR Lesotho OR Malawi OR Mozambique OR Nicaragua OR Nigeria OR Rwanda OR Zambia OR Zimbabwe) AND “sugar” AND (“fortification” OR “fortified” OR “enriched” OR “micronutrient” OR “retin*” OR “iron” OR “mineral” OR “vitamin”) AND (“coverage” OR “survey” OR “study” OR “assessment” OR “evaluation”) AND (“household” OR “woman” OR “women” OR “children” OR “child” OR “population” OR “intake” OR “consumption”) NOT (review) | 1. Sugar AND fortification AND coverage 2. Sugar AND fortified AND coverage |

The search in SciElo was repeated since the above combined strings yielded no hits; the following keywords were used:

| **Food vehicle** | **Search terms for Scielo** |
| --- | --- |
| Salt | salt AND iodine |
| Wheat flour | wheat AND (fortification OR fortified) |
| Vegetable oil | oil AND (fortification OR fortified) |
| Maize meal | maize AND (fortification OR fortified) |
| Rice | rice AND (fortification OR fortified) |
| Sugar | sugar AND (fortification OR fortified) |

# Risk of bias assessment

Risk of bias was assessed using a minimally modified version of a tool initially developed by Hoy et al. (10). The tool was initially developed to assess risk of bias in back pain prevalence studies to be used in the meta-analyses of the Global Burden of Disease (GBD) group and has since been used in several of their publications. The modifications consist of expanding assessment criterion 7 (reliability and validity of instrument) to include a fortification level method assessment; other than this, some examples have been adapted to suit the context of this study.

Table 3 Risk of bias assessment tool

| **Risk of bias items** | | **Risk of bias levels** | **Points**  **scored** |
| --- | --- | --- | --- |
| 1 | Was the study’s target  population a close representation of the national population in relation to relevant variables, e.g. age, sex, occupation? | **Yes (LOW RISK):** The study’s target population was a close representation of the national population. | 0 |
|  |  | **No (HIGH RISK):** The study’s target population was clearly NOT representative of the national population. | 1 |
| 2 | Was the sampling frame a true  or close representation of the target population? | **Yes (LOW RISK):** The sampling frame was a true or close representation of the target population. | 0 |
|  |  | **No (HIGH RISK):** The sampling frame was NOT a true or close representation of the target population. | 1 |
| 3 | Was some form of random  selection used to select the sample, OR, was a census undertaken? | **Yes (LOW RISK):** A census was undertaken, OR, some form  of random selection was used to select the sample (e.g. simple random sampling, stratified random sampling, cluster sampling, systematic sampling). | 0 |
|  |  | **No (HIGH RISK):** A census was NOT undertaken, AND some form of random selection was NOT used to select the sample. | 1 |
| 4 | Was the likelihood of non- response bias minimal? | **Yes (LOW RISK):** The response rate for the study was ≥75%, OR, an analysis was performed that showed no significant difference in relevant demographic characteristics between responders and non- responders. | 0 |
|  |  | **No (HIGH RISK):** The response rate was <75%, and if any analysis comparing responders and non-responders was done, it showed a  significant difference in relevant demographic characteristics between responders and non-responders. | 1 |
| 5 | Were data collected directly  from the subjects (as opposed to a proxy)? | **Yes (LOW RISK):** All data were collected directly from the subjects. | 0 |
|  |  | **No (HIGH RISK):** In some instances, data were collected from a proxy. | 1 |
| 6 | Was an acceptable case  definition used in the study? | **Yes (LOW RISK):** An acceptable case definition was used. | 0 |
|  |  | **No (HIGH RISK):** An acceptable case definition was NOT used. | 1 |
| 7 | Was the study instrument that measured the parameter of interest (e.g. proportion of fortifiable food consumed) shown to have reliability and validity (if necessary)? If applicable, was the method for measuring nutrient content a quantitative one? | **Yes (LOW RISK):** The study instrument had been shown to have reliability and validity (if this was necessary), e.g. test- re- test, piloting, validation in a previous study, etc./if applicable, was a quantitative method used? | 0 |
|  |  | **No (HIGH RISK):** The study instrument had NOT been shown to have reliability or validity (if this was necessary), or qualitative measure only. | 1 |
| 8 | Was the same mode of data  collection? | **Yes (LOW RISK):** The same mode of data collection was used for all subjects. | 0 |
|  |  | No (HIGH RISK): The same mode of data collection was NOT used for all subjects. | 1 |
| 9 | Were the numerator(s) and  denominator(s) for the parameter of interest appropriate | **Yes (LOW RISK):** The paper presented appropriate  numerator(s) AND denominator(s) for the parameter of interest (e.g. the coverage with adequately fortified food) | 0 |
|  |  | No (HIGH RISK): The paper did present numerator(s) AND denominator(s) for the parameter of interest but one or more of these were inappropriate. | 1 |
| 10 | Summary on the overall risk of study bias | LOW RISK | 0-3 |
|  |  | MODERATE RISK | 4-6 |
|  |  | HIGH RISK | 7-9 |

# References

1. UNICEF. UNICEF Data Warehouse [Internet]. [cited 2021 Jun 15]. Available from: https://data.unicef.org/resources/data_explorer/unicef_f/?ag=UNICEF&df=GLOBAL_DATAFLOW&ver=1.0&dq=.NT_IOD_ANY_TS+NT_IOD_ANY_TH..&startPeriod=2016&endPeriod=2021

2. Food Fortification Initiative, Global Alliance for Improved Nutrition, Iodine Global Network, Micronutrient Forum. Global Fortification Data Exchange [Internet]. [cited 2020 Jun 1]. Available from: https://fortificationdata.org/

3. Iodine Global Network. Global Iodine Nutrition Scorecard 2020. Ottowa; 20202.

4. WHO | Micronutrients database [Internet]. World Health Organization,. World Health Organization; 2019 [cited 2019 Oct 1]. Available from: https://www.who.int/vmnis/database/en/

5. MEASURE DHS/ICF International. Statcompiler. Website. 2021.

6. Surveys - UNICEF MICS [Internet]. [cited 2021 Jun 1]. Available from: https://mics.unicef.org/surveys

7. International Household Survey Network. International Household Survey Network [Internet]. [cited 2021 Aug 10]. Available from: http://catalog.ihsn.org/catalog

8. Global Fortification Data Exchange | GFDx – Providing actionable food fortification data all in one place. [Internet]. [cited 2021 Jun 14]. Available from: https://fortificationdata.org/

9. IHSN | International Household Survey Network [Internet]. [cited 2021 Jun 14]. Available from: http://www.ihsn.org/

10. Hoy D, Brooks P, Woolf A, Blyth F, March L, Bain C, Baker P, Smith E, Buchbinder R. Assessing risk of bias in prevalence studies: modification of an existing tool and evidence of interrater agreement. J Clin Epidemiol [Internet]. University of Queensland, Herston Road, Herston, Brisbane, QLD 4006, Australia. damehoy@yahoo.com.au; 2012;65:934–9. Available from: http://europepmc.org/abstract/MED/22742910
