# Supplementary material for: Global Coverage of Mandatory Large-Scale Food Fortification Programs: A Systematic Review and Meta-Analysis
Source: Adv Nutr. 2023 Jul 25;14(5):1197–210. doi: 10.1016/j.advnut.2023.07.004 (PMC10509437; doi:10.1016/j.advnut.2023.07.004)

**Supplementary File 4 – Details about the Meta-Regression**

1. **Variables examined as part of the meta-regression:**

**Human Development Report**

1. Human Development Index (HDI) – Separate indicators by year, 2010 – 2019 (varnames = HDI-2010, HDI-2011, etc)
2. Percent of urban population – Separate indicators by year, 2010 – 2019 (varnames = Urban-pct-2010, Urban-pct-2011, etc)
3. GINI index – One indicator covering 2010 – 2018 (varname = GINI-Coef-2010-2018)
4. GDP per capita in GDP per capita (2017 PPP $) – Separate indicators by year, 2010 – 2019 (varnames = GDP-perCap-2010, GDP-perCap-2011, etc)
5. Employment in agriculture (% of total employment) – Separate indicators by year, 2010 – 2019 (varnames = Employ-Ag-2010, Employ-Ag-2011, etc)
6. Internet users, total (% of population) – Separate indicators by year, 2010 – 2018 (varnames = InternetPCT-2010, InternetPCT-2011, etc)
7. Mobile phone subscriptions (per 100 people) – Separate indicators by year, 2010 – 2018 (varnames = Mobile-2010, Mobile-2011, etc)
8. Skilled labour force (% of labour force) – One indicator covering 2010 – 2019 (varname = SkilledLabor-2010-2019)

**Food Systems Dashboard**

1. # Supermarkets per 100,000 population – One indicator for 2018 (varname = Supermarket-2018) [n=82]
2. # Modern grocery retailers per 100,000 population – One indicator for 2018 (varname = ModGrocery-2018) [n=82]
3. Road Density (km per sq km of land area) - One indicator for 2018 (varname = RoadDensity-2018) [n=80]

**World Bank**

1. Logistics Performance Index – Five indicators for 2010, 2012, 2014, 2016, 2018 (varnames = LPI-2010, LPI-2012, etc)
2. Ease of doing business index – One indicator for 2019 (varname = EODB-2019)
3. Official Development Assistance (ODA) per capita – Separate indicators by year, 2010 – 2019 (varnames = ODA-2010, ODA-2011, etc)
4. Voice and Accountability Index-Estimate, Separate indicators by year, 2010 – 2019 (varnames= VAE2010, VAE2011, etc)
5. Political Stability and Absence of Violence/Terrorism Index-Estimate, Separate indicators by year, 2010 – 2019 (varnames= PVE2010, PVE2011, etc)
6. Government Effectiveness Index-Estimate, Separate indicators by year, 2010 – 2019 (varnames= GEE2010, GEE2011, etc)
7. Regulatory Quality Index-Estimate, Separate indicators by year, 2010 – 2019 (varnames= RQE2010, RQE2011, etc)
8. Rule of Law Index-Estimate, Separate indicators by year, 2010 – 2019 (varnames= RLE2010, RLE2011, etc)
9. Control of Corruption Index-Estimate, Separate indicators by year, 2010 – 2019 (varnames= CCE2010, CCE2011, etc)
10. Log of Gross Domestic Product (GDP) per capita 2019 – Due to a skewed distribution of GDP, the natural log was taken.

**Global Fortification Data Exchange (GFDx)**

1. Year when salt iodization was made mandatory
2. **R results showing Variance Inflation Factor (VIF) results for each regression:**
   1. **National model**

**
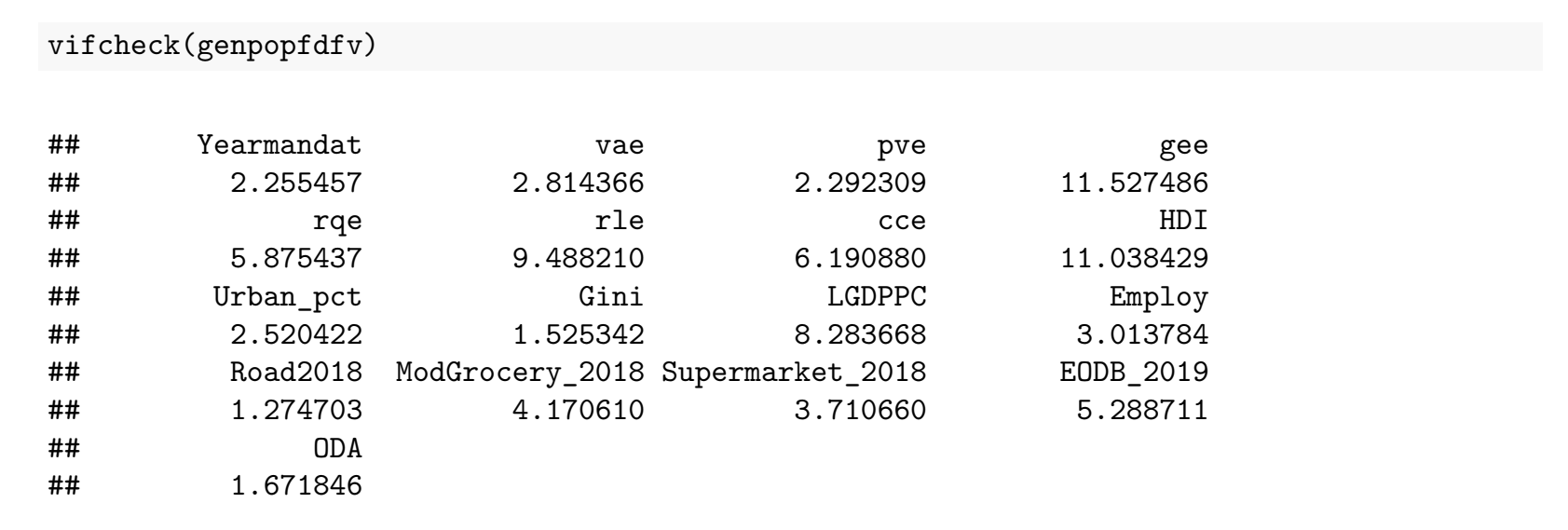
**

- 1. **Urban/Rural Model**


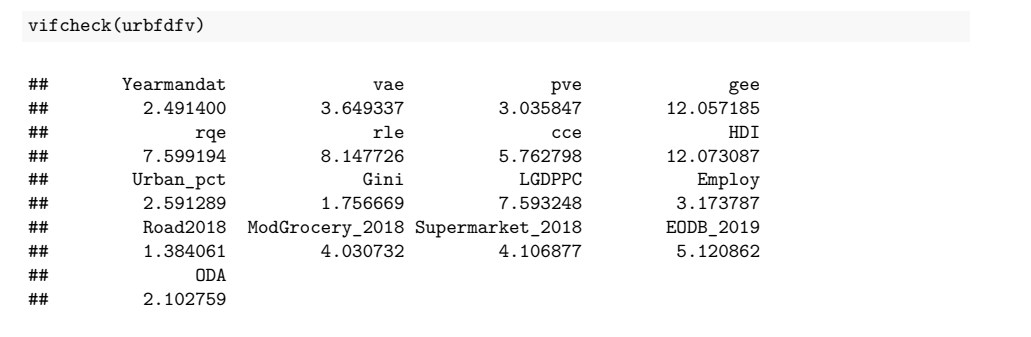


- 1. **Income group model**


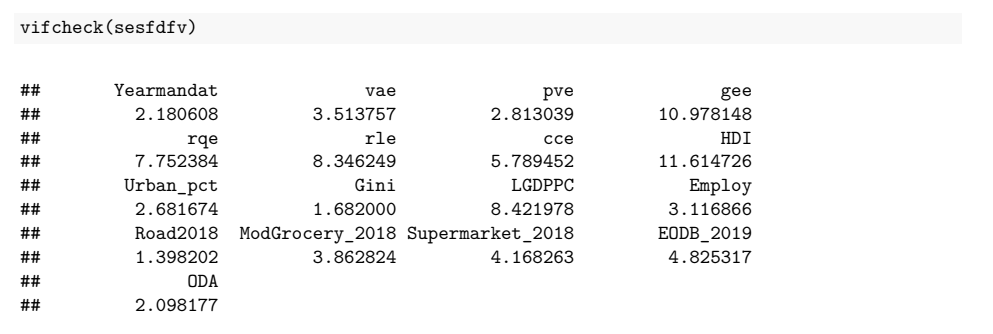

Supplement: Multimedia component2 [file mmc2.docx]
