# Supplementary material for: Global Coverage of Mandatory Large-Scale Food Fortification Programs: A Systematic Review and Meta-Analysis
Source: Adv Nutr. 2023 Jul 25;14(5):1197–210. doi: 10.1016/j.advnut.2023.07.004 (PMC10509437; doi:10.1016/j.advnut.2023.07.004)
Supplement: Multimedia component3 [file mmc3.docx]

**Table 1: Country-specific salt coverage indicators**

| **Country (ref)** | **Survey year** | **Coverage of vehicle (95%CI)** | | **Coverage of fortifiable vehicle (95%CI)** | | **Coverage of fortified vehicle (95%CI)** | | **Coverage of adequately fortified vehicle (95%CI)** | | | **Comment** |
| --- | --- | --- | --- | --- | --- | --- | --- | --- | --- | --- | --- |
|  |  | **N** | **% (95%CI)** | **N** | **% (95%CI)** | **N** | **% (95%CI)** | **N** | **% (95%CI)** | **Cutoff** |  |
| Afghanistan (1) | 2017 | 2474 | 100.0 (100.0, 100.0) | 2474 | 100.0 (100.0, 100.0) | 2474 | 22.1 (10.6, 33.6) | -- | -- | -- | Sampling frame: households were only selected, if at least one child <5 years lived there;  Fortification measure: samples were collected and analyzed from markets and at household level, the brand name was asked for and during analysis, the corresponding nutrient content was allocated;  Urban/rural sub-groups: Urban/rural estimates have been recalculated based on weights from population figures by province, since in the report Kabul and other cities were presented in separate categories. |
| Albania (2) | 2018 | 15823 | 99.4 (99.2, 99.6) | -- | -- | 15534 | 64.6 (62.2, 66.9) | -- | -- | -- | Vehicle consumption: the coverage of food vehicle was estimated based on household presence of salt at the time of survey |
| Algeria (3) | 2019 | 29873 | 99.6 (99.5, 99.7) | -- | -- | 29793 | 89.1 (88.0, 90.2) | -- | -- | -- |  |
| Angola (4) | 2019 | -- | -- | -- | -- | 2225 | 74.3 (--, --) | 2225 | 29.2 (--, --) | 15-40 | Sampling frame: households were only selected, if at least one woman of reproductive age lived there. |
| Armenia (5) | 2016 | -- | -- | -- | -- |  | -- | -- | -- | 25-65 | Sampling frame: Only urban/peri-urban clusters were included in the survey; therefore, no national estimate provided, but only for urban. |
| Azerbaijan (6) | 2013 | 3927 | 99.7 (99.2, 99.8) | -- | -- | 3764 | 93.8 (92.5, 94.8) | -- | -- |  | Vehicle consumption: the coverage of food vehicle was estimated based on household presence of salt at the time of survey |
| Bahrain (7) | 2014 |  |  | -- | -- | 1051 | 57.7 (--, --) | 1051 | 36.9 (--, --) | 15-40 | Sampling frame: households were only selected, if at least one child 6-12 years lived there. |
| Bangladesh (8) | 2019 | 61230 | 99.4 (99.3, 99.5) | -- | -- | 61217 | 76.0 (75.4, 76.6) | -- | -- |  |  |
| Belize (9) | 2018 |  |  | -- | -- | -- | -- | 9804 | 51 (--, --) | 15-40 | Sampling: A rapid population-based survey to gather initial data for Caribbean countries with no data on iodized status. Not necessarily nationally representative. |
| Benin (10) | 2018 | 14156 | 94.6 (94.1, 95.0) | -- | -- | 14044 | 84.7 (83.8, 85.7) | -- | -- | -- |  |
| Bolivia (Plurinational State of) (11) | 2017 | 14655 | 93.8 (93.3, 94.3) | -- | -- | 14607 | 85.7 (85.0, 86.4) | -- | -- | -- |  |
| Burkina Faso (12) | 2014 | 3786 | 96.9 (95.7, 96.9) | -- | -- | 3274 | 82.4 (80.4, 84.1) | 3339 | 23.0 (21.0, 25.2) | ≥15 | Vehicle consumption: the coverage of food vehicle was estimated based on household presence of salt at the time of survey |
| Burundi (13) | 2018 | 541 | 93.5 (91.0, 95.0) | -- | -- | 540 | 21.3 (18.0, 25.0) | 541 | 52.7 (49.0, 67.0) | 15-40 |  |
| Cambodia (14) | 2015 | 15812 | 98.9 (98.7, 99.1) | -- | -- | 15804 | 68.2 (66.3, 69.9) | -- | -- | -- |  |
| Cameroon | 2019 | 11710 | 93.6 (93.0, 94.1) | -- | -- | 11658 | 90.7 (89.7, 91.5) | -- | -- | -- |  |
| Cape Verde (15) | 2010 | -- | -- | -- | -- | 2520 | 91.9 (--, --) | 2520 | 37.6 (--, --) | 20-40 | Sampling frame: school-based sampling frame where school-age children were randomly selected. |
| Central African Republic (16) | 2019 | 8132 | 80.4 (79.1, 81.6) | -- | -- | 8070 | 76.0 (74.5, 77.3) | -- | -- | -- |  |
| Chad (17) | 2019 | 18941 | 95.4 (94.9, 95.9) | -- | -- | 18838 | 65.0 (63.3, 66.6) | -- | -- | -- |  |
| China (18) | 2017 |  |  | -- | -- | 78470 | 95.37 (90.0, 97.9) | -- | -- | -- |  |
| Congo (19) | 2015 | 12810 | 93.2 (92.5, 93.9) | -- | -- | 12738 | 91.1 (90.2, 91.9) | -- | -- | -- |  |
| Congo-DRC (20) | 2018 | 20792 | 90.3 (--, --) | -- | -- | 20732 | 84.7 (--, --) | -- | -- | -- | Vehicle consumption: the coverage of food vehicle was estimated based on household presence of salt at the time of survey |
| Cote d'Ivoire (21) | 2016 | 11873 | 89.3 (88.2, 90.3) | -- | -- | 11786 | 79.7 (78.1, 81.2) | -- | -- | -- |  |
| Ecuador (22) | 2017 | -- | -- | -- | -- | 1119 | 99.6 (--, --) | 1119 | 97.9 (--, --) | 15-40 | Sampling frame: school-based sampling frame where school-age children were randomly selected. |
| Egypt (23) | 2015 | -- | -- | -- | -- | 2914 | 92.5 (--, --) | 2914 | 74.7 (--, --) | ≥15 | Sampling frame: School-based sampling frame; selected children brought salt from their homes. |
| El Salvador (24) | 2012 | -- | -- | -- | -- | 424 | 73.8 (--, --) | 424 | 66.5 (--, --) | 20-100 | Sampling frame: In selected households, the age range of selected school-age children was 7-9 years. |
| Eswatini (25) | 2015 | 4860 | 94.9 (94.0, 95.7) | -- | -- | 4845 | 90.4 (89.2, 91.4) | -- | -- | -- |  |
| Ethiopia (26,27) | 2016; 2015 | 16650 | 95.9 (95.3, 96.4) | -- | -- | 16619 | 85.6 (83.7, 87.4) | 12484 | 25.8 (--, --) | ≥15 | Data source: adequately fortified vehicle from (27) |
| Fiji (28) | 2021 | -- | -- | -- | -- | 5467 | 98.9 (--, --) | -- | -- | -- | Based on preliminary report |
| Gabon (29) | 2012 | 9644 | 91.8 (90.5, 92.9) | -- | -- | 9595 | 89.5 (88.0, 90.8) | -- | -- | -- |  |
| Gambia (30,31) | 2018;  2020 | 6549 | 87.3 (--, --) | -- | -- | 5630 | 77.0 (--, --) | 843 | 10.8 (8.0, 14.5) | ≥15 | Data source: adequately fortified vehicle from (31) |
| Georgia (32) | 2017 | -- | -- | -- | -- | 833 | 100.0 (--, --) | 833 | 86.4 (--, --) | 25-55 |  |
| Ghana (33,34) | 2015;  2015 | 12886 | 93.4 (92.6, 94.0) | -- | -- | 12764 | 68.9 (67.1, 70.6) | 1563 | 29.3 (25.3, 33.6) | ≥15 | Data source: adequately fortified vehicle from (34) |
| Guatemala (35) | 2016 | -- | -- | -- | -- | 553 | 68.4 (--, --) | 553 | 30.7 (--, --) | 15-39.9 |  |
| Guinea (36) | 2019 | 7912 | 95.4 (94.6, 96.1) | -- | -- | 7855 | 52.6 (48.8, 56.4) | -- | -- | -- |  |
| Guinea-Bissau (37) | 2019 | 7379 | 96.2 (95.6, 96.8) | -- | -- | 7333 | 32.6 (30.1, 35.2) | -- | -- | -- |  |
| Haiti (38) | 2017 | 13405 | 92.6 (91.8, 93.3) | -- | -- | 13398 | 7.5 (6.5, 8.7) | -- | -- | -- |  |
| India (39) | 2019 | -- | -- | -- | -- | 21406 | 92.4 (--, --) | 21406 | 76.3 (74.1, 78.5) | ≥15 | Fortification measure: Adequately iodised salt measure has two cut-offs (15-30 ppm and ≥15 ppm); the one more commonly used internationally was used. |
| Iran (40) | 2014 | -- | -- | -- | -- | 1800 | 98.0 (--, --) | 1800 | 63.0 (--, --) | 20-40 | Salt sampling: salt samples collected from school age children who were asked to bring salt samples from their households; Fortification analysis: rapid test kits used and titration done on sub-sample for iodine analysis. Sub-sample selection procedure unclear |
| Jordan (41) | 2010 | -- | -- | -- | -- | 4426 | 96.3 (--, --) | -- | -- | -- |  |
| Kazakhstan (42) | 2016 | 2004 | 99.9 (99.8, 100.0) | 2004 | 99.9 (99.8, 100) | 2004 | 88.4 (87, 89.8) | 2004 | 80.5 (78.7, 82.2) | 25-65 |  |
| Kenya (43) | 2015 | 36277 | 95.2 (94.8, 95.5) | -- | -- | 35872 | 94.6 (94.2, 95.0) | -- | -- | -- |  |
| Kiribati (44) | 2019 | 3068 | 80.2 (78.2, 81.9) | -- | -- | 3048 | 76.9 (74.9, 78.8) | -- | -- | -- |  |
| Kyrgyzstan (45) | 2019 | 6968 | 99.1 (98.8, 99.4) | -- | -- | 6901 | 98.7 (98.3, 99.0) | -- | -- | -- |  |
| Lao PDR (46) | 2018 | 22285 | 98.7 (98.5, 98.9) | -- | -- | 22278 | 93.6 (92.7, 94.3) | -- | -- | -- |  |
| Lesotho (47) | 2015 | 9402 | 93.5 (92.7, 94.3) | -- | -- | 7239 | 85.0 (83.5, 86.4) | -- | -- | -- |  |
| Liberia (48) | 2020 | 9068 | 87.9 (--, --) | -- | -- | 9068 | 86.4 (--, --) | -- | -- | -- |  |
| Macedonia (49) | 2016 | -- | -- | -- | -- | 1114 | 98.3 (--, --) | 1114 | 87.1 (--, --) | 15-30 |  |
| Madagascar (50) | 2016 | -- | -- | -- | -- | 1140 | 84.1 (--, --) | 1140 | 21.3 (15.5, 27.1) | ≥15 | Analysis: Socioeconomic status data collected; however, data was not analyzed |
| Malawi (51,52) | 2016;  2016 | 26361 | 86.9 (86.2, 87.5) | -- | -- | 26014 | 77.7 (76.4, 78.9) | 1943 | 41.0 (35.9, 46.2) | 15-40 | Data source: adequately fortified vehicle from (52) |
| Mali (53) | 2019 | 9090 | 100.0 (100.0, 100.0) | -- | -- | 9083 | 89.4 (87.3, 91.1) | -- | -- | -- |  |
| Mauritania (54) | 2016 | 11748 | 96.4 (95.8, 96.9) | -- | -- | 11437 | 7.6 (6.5, 8.9) | -- | -- | -- |  |
| Republic of Moldova (55) | 2012 | 11353 | 99.2 (98.9, 99.3) | -- | -- | 10719 | 58.0 (56.0, 59.9) | -- | -- | -- |  |
| Mongolia (56) | 2019 | 13793 | 99.3 (98.9, 99.5) | -- | -- | 13685 | 75.1 (73.7, 76.5) | -- | -- | -- |  |
| Morocco (57) | 2019 | 3118 | 100.0 (--, --) | 3118 | 89.0 (87.9, 90.1) | 1963 | 74.3 (72.4, 76.2) | 1963 | 7.5 (6.4, 8.7) |  | Analysis: Socioeconomic status data was presented in tertile. |
| Mozambique (58) | 2011 | 13919 | 94.8 (94.3, 95.3) | -- | -- | 13819 | 42.5 (40.6, 44.4) | -- | -- | -- |  |
| Myanmar (59) | 2018 |  |  | -- | -- | 27337 | 85 (82.5, 87.1) | 27337 | 36.8 (34.5, 39.1) | ≥15 | Analysis: Food security data presented as mildly, moderately, and severely food insecure. Not dichotomous (food secure or insecure) |
| Namibia (60) | 2014 | 9780 | 95.9 (95.4, 96.3) | -- | -- | 9612 | 73.6 (72.1, 75.1) | -- | -- | -- |  |
| Nepal (61) | 2017 | 11040 | 99.2 (99.0, 99.4) | -- | -- | 11020 | 94.2 (93.1, 95.1) | -- | -- | -- |  |
| Niger (62) | 2018 |  |  | -- | -- | 5853 | 81.8 (--, --) | -- | -- | -- | Sampling frame: Though the data presented is nationally representative, a few high-risk EAs were excluded from data collection. |
| Nigeria (63) | 2019 | 40427 | 96.2 (95.9, 96.5) | -- | -- | 39525 | 93.3 (92.8, 93.8) | -- | -- | -- |  |
| Oman (64) | 2014 | -- | -- | -- | -- | -- | 87.7 (--, --) | -- | -- | -- | Not included in data analysis, as only a point estimate without any sample size estimate was available |
| Palestine (State of) (65) | 2020 | 9325 | 98.5 (98.2, 98.8) | -- | -- | 9276 | 95.7 (95.0, 96.2) | -- | -- | -- |  |
| Papua New Guinea (66) | 2017 | 16021 | 77.3 (--, --) | -- | -- |  |  | 16021 | 60.7 (--, --) | ≥15 |  |
| Paraguay (67) | 2019 |  |  | -- | -- | 3700 | 100.0 (--, --) | 3700 | 75.8 (--, --) | 20-40 | Vehicle consumption: Coverage on vehicle consumption presented/reported annually. Methodology is unclear on this |
| Peru (68) | 2020 | 34971 | 95.8 (95.4, 96.2) | -- | -- | 33256 | 90.8 (90.2, 91.4) | -- | -- | -- |  |
| Philippines (69) | 2018 | -- | -- | -- | -- | -- | 57.0 (--, --) | -- | -- | -- | Not included in data analysis, as only a point estimate with a weighted sample size was available |
| Qatar (70) | 2014 |  |  | -- | -- | 1006 | 87.1 (--, --) | 1006 | 74.7 (--, --) | 15-40 | Sampling frame: School-based sampling frame; selected children brought salt from their homes. Fortification measure: data on non-iodized salt not presented. |
| Rwanda (71) | 2015 | 12655 | 90.8 (90.2, 91.3) | -- | -- | 12647 | 90.5 (89.9, 91.1) | -- | -- | -- |  |
| Sao Tome and Principe (72) | 2020 | 3422 | 91.6 (90.3, 92.8) | -- | -- | 3346 | 88.6 (87.0, 90.0) | -- | -- | -- |  |
| Saudi Arabia (73) | 2012 |  |  | -- | -- | 4242 | 68.7 (67.3, 70.1) | 775 | 69.8 (68.4, 71.2) | ≥15 | Fortification measure: Adequately iodized salt nationally weighted, however, percentage adequately iodized is greater than percentage iodized. |
| Senegal (74) | 2019 | 4592 | 91.3 (89.9, 92.5) | -- | -- | 4586 | 65.1 (62.5, 67.6) | -- | -- | -- |  |
| Sierra Leone (75) | 2020 | 13399 | 91.6 (90.8, 92.4) | -- | -- | 13284 | 82.2 (80.7, 83.6) | -- | -- | -- |  |
| Solomon Islands (76) | 2016 | 4951 | 90.1 (88.2, 91.7) | -- | -- | 4903 | 88.4 (86.5, 90.1) | -- | -- | -- |  |
| Somalia (77) | 2019 | 2172 | 94.0 (--, --) | -- | -- | 2172 | 20.4 (--, --) | 1271 | 7.0 (3.8, 12.5) | ≥15 |  |
| South Africa (78) | 2017 | 2804 | 93.5 (92.1, 94.7) | -- | -- | 2653 | 91.1 (89.6, 92.5) | -- | -- | -- |  |
| Sri Lanka (79) | 2017 | 27210 | 96.2 (95.9, 96.5) | -- | -- | 27210 | 91.6 (91.2, 92.0) | -- | -- | -- |  |
| Sudan (80) | 2015 | 16673 | 95.2 (94.5, 95. 8) | -- | -- | 16557 | 34.4 (32.3, 36.6) | -- | -- | -- |  |
| Tajikistan (81) | 2018 | 7843 | 99.6 (99.4, 99.7) | -- | -- | 7838 | 91.4 (90.2, 92.4) | -- | -- | -- |  |
| Tanzania (82) | 2018 | 8864 | 90.5 (--, -- | -- | -- | 8864 | 86.2 (82.2, 90.1) | 8864 | 55.4 (59.3, 63.1) | ≥15 |  |
| Thailand (83) | 2020 | 35577 | 89.9 (88.9, 90.7) | -- | -- | 35020 | 84.1 (83.0, 85.2) | -- | -- | -- |  |
| Togo (84) | 2018 | 7916 | 95.9 (95.2, 96.5) | -- | -- | 7866 | 80.5 (79.3, 81.7) | -- | -- | -- |  |
| Tunisia (85) | 2012 |  |  | -- | -- | 1560 | 93.8 (91.6, 95.4) | 1560 | 55.8 (50.5, 61.1) | 25-45 | Response rate: 100% response rate achieved but due to the replacement of non-responding clusters |
| Turkmenistan (86) | 2016 | 5860 | 99.9 (99.7, 100.0) | -- | -- | 5856 | 99.7 (99.5, 99.9) | -- | -- | -- |  |
| Uganda (87) | 2017 | 19588 | 91.9 (91.3, 92.5) | -- | -- | 19465 | 91.3 (90.7, 91.9) | -- | -- | -- |  |
| Uzbekistan (88) | 2017 | 3791 | 98 (97.5, 98.4) | -- | -- | 3413 | 56.1 (52.3, 60.1) | 3413 | 36.4 (34.1, 38.7) | ≥15 |  |
| Viet Nam (89) | 2011 | 11596 | 97.6 (97.2, 97.9) | -- | -- | 11556 | 60.9 (58.5, 63.2) |  |  |  |  |
| Yemen (90) | 2015 | 3658 | 94.0 (--, --) | -- | -- | 3783 | 93.7 (--, --) | 3783 | 3.5 (--, --) | ≥15 | Sampling frame: Almost all data presented is on children 6-12 years living in selected households. Vehicle consumption: All assessments of salt done at the household level; however, a sub-sample of children 6-12 years were later selected for urinary analysis |
| Zambia (91) | 2014 | 15729 | 93.1 (92.5, 93.7) | -- | -- | 14556 | 88.4 (87.6, 89.2) | -- | -- | -- |  |
| Zimbabwe (92) | 2019 | 11091 | 97.0 (96.6, 97.3) | -- | -- | 11023 | 83.8 (82.9, 84.7) | -- | -- | -- |  |

**Table 2: Country-specific wheat flour coverage indicators**

| **Country (ref)** | **Survey year** | **Coverage of vehicle (95%CI)** | | **Coverage of fortifiable vehicle (95%CI)** | | **Coverage of fortified vehicle (95%CI)** | | **Coverage of adequately fortified vehicle (95%CI)** | | | **Comment** |
| --- | --- | --- | --- | --- | --- | --- | --- | --- | --- | --- | --- |
|  |  | **N** | **% (95%CI)** | **N** | **% (95%CI)** | **N** | **% (95%CI)** | **N** | **% (95%CI)** | **Cutoff** |  |
| Afghanistan (1) | 2017 | 2474 | 91.6 (86.0, 97.3) | 2474 | 49.7 (34.7, 64.7) | 2474 | 18.6 (10.8, 26.4) |  |  |  | Sampling frame: households were only selected, if at least one child <5 years lived there; Fortification measure: samples were collected and analyzed from markets and at household level, the brand name was asked for and during analysis, the corresponding nutrient content was allocated;  Urban/rural sub-groups: Urban/rural estimates have been recalculated based on weights from population figures by province, since in the report Kabul and other cities were presented in separate categories. |
| El Salvador (24) | 2012 | -- | -- | -- | -- | 209 | 85.2 (--, --) | 209 | 42.1 (--, --) | 55-84.9 | Sampling frame: In selected households, the age range of selected school-age children was 7-9 years; Fortification measure: Bread and pasta separately collected and tested. |
| Fiji (28) | 2010 | 869 | 95.1 (--, --) | -- | -- | -- | -- | -- | -- | -- | Sampling frame: households were only selected, if at least one woman of reproductive age lived there; Fortification measure: samples were collected and analyzed from the markets. |
| Gambia (31) | 2018 | 440 | 65.3 (54.4, 74.8: | -- | -- | -- | -- | -- | -- | -- |  |
| Ghana (93) | 2017 | 2082 | 81.5 (75.6, 86.3) | -- | -- | -- | -- | -- | -- | -- |  |
| Guatemala (35) | 2016 | 2375 | 92.2 (--, --) |  |  | 96 | 100 (--, --) | 96 | 44.8 (--, --) | ≥55 |  |
| Jordan (94) | 2010 |  |  | -- | -- | 1737 | 44.1 (40.2, 48) |  |  |  | Fortification measure: iron analyzed through iron spot test; Iron tested from bread and not flour |
| Kazakhstan (42) | 2016 | 2004 | 99.5 (99.2, 99.8) | 2004 | 99.4 (99.1, 99.7) | 2004 | 40.7 (38.6, 42.9) | 2004 | 25.1 (23.2, 27.0) | 45-65 |  |
| Malawi (52) | 2016 | 2090 | 61.2 (55, 67.5) | -- | -- | -- | -- | -- | -- | -- | Vehicle consumption: Coverage of food vehicle based on households purchase of the food vehicle |
| Mongolia (95) | 2010 | -- | -- | -- | -- | 930 | 2.5 (1.4, 4.2) | -- | -- | -- |  |
| Morocco (57) | 2019 | 3118 | 100.0 (--, --) | 3118 | 70.2 (68.6, 71.8) |  |  |  |  |  | Analysis: Socioeconomic status data was presented in tertile. |
| Mozambique (96) | 2013 | -- | -- | -- | -- | -- | -- | -- | -- | -- |  |
| Nepal (97) | 2016 | -- | -- | -- | -- | -- | -- | 943 | 36.8 (32.6, 41.2) | ≥60 | Vehicle consumption: the coverage of food vehicle was estimated based on household presence of wheat flour at the time of survey. If a household has more than one type of wheat flour brand, a sample from the most frequently consumed is collected. |
| Senegal (98) | 2013 | 1910 | 81.8 (76.2, 86.6) | 1910 | 81.5 (75.5, 86.4) | 1910 | 51.2 (44.7, 57.2) | -- | -- | -- |  |
| Sierra Leone (99) | 2013 | 1306 | 49.4 (43, 55.9) | 645 | 49.4 (43, 55.9) | -- | -- | -- | -- | -- | Fortification measure: Vehicle fortification not measured. Bread was analyzed instead of wheat flour for fortification levels. |
| Solomon Islands (100) | 2013 | 4478 | 86.6 (--, --) | 4478 | 86.6 (--, --) | 4478 | 86.6 (--, --) | -- | -- | -- | Vehicle consumption: Coverage of wheat flour based on household's purchase of wheat flour |
| Tanzania (101) | 2015 | 1036 | 51.5 (44.5, 58.5) | 1036 | 50.5 (43.3, 57.7) | 1036 | 33.1 (27.5, 38.8) | -- | -- | -- | Urban/rural sub-groups: Urban/rural disaggregation excludes Zanzibar, but included in 'national' estimate |
| Uganda | 2015 | 949 | 11.2 (7.7, 14.7) | 949 | 10.6 (6.8, 13.6) | 949 | 8.5 (5.7, 11.4) | -- | -- | -- |  |
| Uzbekistan (88) | 2017 | 3874 | 99.4 (99.1, 99.6) |  |  |  |  | 3366 | 29.9 (25.8, 29.9) | ≥30 | Fortification measure: Bread was analyzed instead of wheat flour for fortification levels. |
| Zimbabwe (102) | 2019 | 1953 | 93.0 (--, --) | -- | -- | -- | -- | -- | -- | -- | Sampling frame: Method used in the selection of the 10 regions unclear. Survey: Emergency nutrition assessment using SMART Methodology. Fortification measure: Fortification results on fortified was based on the presence of a fortification logo on the vehicle. |

**Table 3: Country-specific oil coverage indicators**

| **Country (ref)** | **Survey year** | **Coverage of vehicle (95%CI)** | | **Coverage of fortifiable vehicle (95%CI)** | | **Coverage of fortified vehicle (95%CI)** | | **Coverage of adequately fortified vehicle (95%CI)** | | | **Comment** |
| --- | --- | --- | --- | --- | --- | --- | --- | --- | --- | --- | --- |
|  |  | **N** | **% (95%CI)** | **N** | **% (95%CI)** | **N** | **% (95%CI)** | **N** | **% (95%CI)** | **Cutoff** |  |
| Afghanistan (1) | 2017 | 2474 | 100.0 (100.0, 100.0) | 2474 | 98.8 (97.9, 99.8) | 2474 | 30.1 (24, 36.2) | -- | -- | -- | Sampling frame: households were only selected, if at least one child <5 years lived there; Fortification measure: samples were collected and analyzed from markets and at household level, the brand name was asked for and during analysis, the corresponding nutrient content was allocated;  Urban/rural sub-groups: Urban/rural estimates have been recalculated based on weights from population figures by province, since in the report Kabul and other cities were presented in separate categories. |
| Bangladesh (103) | 2010 | -- | -- | 12240 | 90.6 (--,--) | -- | -- | -- | -- | -- | Analysis: Though SES was analyzed, only four sub-group categories were presented. Therefore, SES data was not used. |
| Gambia (31) | 2018 | 1004 | 98.6 (97.1, 99.3) | -- | -- | -- | -- | -- | -- | -- |  |
| Ghana (93) | 2017 | 2037 | 70.3 (65.1, 75.0) | -- | -- | -- | -- | 2191 | 55.6 (37.0, 52.0) | ≥10 |  |
| Malawi (52) | 2016 | 2090 | 76.5 (70.3, 82.6) | -- | -- | -- | -- | 1441 | 11.9 (7.2, 16.7) | ≥20 | Vehicle measure: Fortification results on fortified was based on the presence of a fortification logo/label on the vehicle. |
| Mozambique (96) | 2013 |  |  | -- | -- | -- | -- | -- | -- | -- |  |
| Pakistan (104) | 2017 | 2104 | 100.0 (100.0, 100.0) | 2104 | 99.5 (98.8, 100.0) | 2104 | 28.8 (24.0, 33.5) | -- | -- | -- | Analysis: 'National' estimate has been recalculated based on weights from population figures by province |
| Senegal (98) | 2013 | 1910 | 97.8 (96.3, 99.1) | 1910 | 95.0 (92.9, 96.8) | 1910 | 34.1 (29.1, 40.7) | -- | -- | -- | Also contains data from (105) |
| Sierra Leone (99) | 2013 | 1405 | 49.1 (41.8, 56.3) | 690 | 49.1 (41.8, 56.3) |  |  | -- | -- | -- | Fortification measure: Vehicle fortification not measured |
| Tanzania (101) | 2015 | 1036 | 96.2 (93.2, 99.2) | 1036 | 92.6 (89.0, 96.3) | 1036 | 53.6 (46.4, 60.8) | -- | -- | -- | Urban/rural sub-groups: Urban/rural disaggregation excludes Zanzibar, but included in 'national' estimate |
| Uganda (106) | 2015 | 949 | 89.9 (85.9, 94.0) | 949 | 89 (84.7, 93.2) | 949 | 54.4 (48.3, 60.4) | -- | -- | -- |  |
| Zimbabwe (102) | 2019 | 1953 | 89.0 (--, --) | -- | -- | -- | -- | -- | -- | -- | Sampling frame: Method used in the selection of the 10 regions unclear. Survey: Emergency nutrition assessment using SMART Methodology. Fortification measure: Fortification results on fortified was based on the presence of a fortification logo on the vehicle. |

**Table *4*: Country-specific *maize flour* coverage indicators**

| **Country (ref)** | **Survey year** | **Coverage of vehicle (95%CI)** | | **Coverage of fortifiable vehicle (95%CI)** | | **Coverage of fortified vehicle (95%CI)** | | **Coverage of adequately fortified vehicle (95%CI)** | | | **Comment** |
| --- | --- | --- | --- | --- | --- | --- | --- | --- | --- | --- | --- |
|  |  | **N** | **% (95%CI)** | **N** | **% (95%CI)** | **N** | **% (95%CI)** | **N** | **% (95%CI)** | **Cutoff** |  |
| El Salvador (24) | 2012 |  |  |  |  | 82 | 91.5 (--, --) | 82 | 91.5 (83.2, 96.5) | ≥40 | Sampling frame: In selected households, the age range of selected school-age children was 7-9 years. |
| Guatemala (107) | 2019 | 2490 | 31.7 (--, --) | -- | -- | -- | -- | -- | -- | -- | Vehicle analysis: Methods section refers to analysis of the food vehicle, however, food analysis information missing in the report |
| Malawi (52) | 2016 | 2090 | 90.5 (86.8, 94.1) | -- | -- | -- | -- | -- | -- | -- | Vehicle consumption: Coverage of food vehicle based on households purchase of the food vehicle |
| Mozambique (96) | 2013 |  |  | -- | -- | -- | -- | -- | -- | -- |  |
| Tanzania (101) | 2015 | 1036 | 93.0 (89.7, 96.4) | 1036 | 36.6 (29.2, 44) | 1036 | 2.5 (1.3, 3.7) | -- | -- | -- | Urban/rural sub-groups: Urban/rural disaggregation excludes Zanzibar, but included in 'national' estimate |
| Uganda (106) | 2015 | 949 | 91.8 (87.7, 96.0) | 949 | 42.4 (32.7, 52.1) | 949 | 6.7 (3.3, 9.7) | -- | -- | -- |  |
| Zimbabwe (102) | 2019 | 1953 | 27.0 (--, --) | -- | -- | -- | -- | -- | -- | -- | Sampling frame: Method used in the selection of the 10 regions unclear. Survey: Emergency nutrition assessment using SMART Methodology. Fortification measure: Fortification results on fortified was based on the presence of a fortification logo on the vehicle. |

**Table 5: Country-specific *sugar* coverage indicators**

| **Country (ref)** | **Survey year** | **Coverage of vehicle (95%CI)** | | **Coverage of fortifiable vehicle (95%CI)** | | **Coverage of fortified vehicle (95%CI)** | | **Coverage of adequately fortified vehicle (95%CI)** | | | **Comment** |
| --- | --- | --- | --- | --- | --- | --- | --- | --- | --- | --- | --- |
|  |  | **N** | **% (95%CI)** | **N** | **% (95%CI)** | **N** | **% (95%CI)** | **N** | **% (95%CI)** | **Cutoff** |  |
| El Salvador (24) | 2012 | -- | -- | -- | -- | 423 | 96.0 (--, --) | 423 | 75.0 (--, --) | 5-19.9 | Sampling frame: In selected households, the age range of selected school-age children was 7-9 years. |
| Guatemala (35) | 2016 | -- | -- | -- | -- | 258 | 39.2 (--, --) | 258 | 20.6 (--, --) | 6.0-12.0 |  |
| Malawi (52) | 2016 | 2090 | 74.2 (67.6, 80.7) | -- | -- | -- | -- | 1567 | 58.2 (52.3, 64.2) | ≥4 | Vehicle measure: Fortification results on fortified was based on the presence of a fortification logo/label on the vehicle. |
| Mozambique (96) | 2013 | -- | -- | -- | -- | -- | -- | -- | -- | -- |  |
| Zimbabwe (102) | 2019 | 1953 | 64.0 (--, --) | -- | -- | -- | -- | -- | -- | -- | Sampling frame: Method used in the selection of the 10 regions unclear. Survey: Emergency nutrition assessment using SMART Methodology. Fortification measure: Fortification results on fortified was based on the presence of a fortification logo on the vehicle. |

**Table 6: Country-specific *rice* coverage indicators**

| **Country (ref)** | **Survey year** | **Coverage of vehicle (95%CI)** | | **Coverage of fortifiable vehicle (95%CI)** | | **Coverage of fortified vehicle (95%CI)** | | **Coverage of adequately fortified vehicle (95%CI)** | | | **Comment** |
| --- | --- | --- | --- | --- | --- | --- | --- | --- | --- | --- | --- |
|  |  | **N** | **% (95%CI)** | **N** | **% (95%CI)** | **N** | **% (95%CI)** | **N** | **% (95%CI)** | **Cutoff** |  |
| Papua New Guinea (66) | 2017 | 16021 | 66.0 (--,--) | -- | -- | -- | -- | -- | -- | -- |  |
| Solomon Islands (100) | 2013 | 4478 | 95.6 (--,--) | 4478 | 95.6 (--,--) | 4478 | 95.6 (--,--) | -- | -- | -- | Because all rice is imported and tested at point of importation (and complied with standards), the estimate of ‘fortified vehicle’ has been extrapolated. |

**References**

1. Global Alliance for Improved Nutrition. REPORT ( FACT ) SURVEY IN AFGHANISTAN , 2017 [Internet]. 2018. Available from: https://www.gainhealth.org/resources/reports-and-publications/fortification-assessment-coverage-toolkit-fact-survey

2. Institute of Public Health (IPH), ICF. Albania Demographic and Health Survey 2017-18 [Internet]. Triana, Albania: INSTAT, IPH, and ICF; 2018. Available from: http://dhsprogram.com/pubs/pdf/FR348/FR348.pdf

3. Ministère de la Santé de la P et de la RH, UNICEF. Enquête par grappes à indicateurs multiples [MICS] 2019 [Internet]. 2021. Available from: https://mics-surveys-prod.s3.amazonaws.com/MICS6/Middle East and North Africa/%0AAlgeria/2018-2019/Survey findings/Algeria 2018-19 MICS_French.pdf

4. IODINE IN ANGOLA: AVAILABILITY AND QUALITY OF CONSUMPTION 2019 [Internet]. 2019. Available from: file:///C:/Users/MisterDee/Dropbox/GAIN_LSFF_meta-analysis/14_Data & analysis/7_Full texts/77_Inventarized reports/AGO_2 2019 COV salt.pdf

5. Hutchings N, Aghajanova E, Baghdasaryan S, Qefoyan M, Sullivan C, He X, Manoukian M, Gerasimov G, Braverman L, Bilezikian JP. Iodine nutrition in Armenia: Preliminary Report [Internet]. 2016. Available from: file:///C:/Users/MisterDee/Dropbox/GAIN_LSFF_meta-analysis/14_Data & analysis/7_Full texts/77_Inventarized reports/ARM_2 2016 COV salt.pdf

6. UNICEF, Ministry of Health Azerbaijan, GroundWork. AZERBAIJAN NUTRITION SURVEY (AzNS), 2013 [Internet]. 2013. Available from: https://groundworkhealth.org/wp-content/uploads/2015/06/UNICEF-2013_Azerbaijan-National-Nutrition-Survey_report_eng_compressed.pdf

7. Al-Jawaldeh A, Gharib N, Sharief I, Salehi S Al, Amer M Al. Prevalence and Risk Factors of Iodine Deficiency Among School Children (6-12) years in Kingdom of Bahrain. 2014.

8. Bangladesh Bureau of Statistics. Bangladesh Multiple Cluster Indicator Survey [Internet]. 2019. Available from: https://mics.unicef.org/surveys

9. Zimmermann MB, Stoffel DN, Giorgetti A, Grajeda R, Xuereb G, Smuts M, Kupka R. Caribbean Island Urinary Iodine Survey 2018 ( CRUISE ). 2018.

10. Institut National de la Statistique et de l’Analyse Économique (INSAE), ICF. République Du Bénin Ciquième Enquête Démographique et de Santé au Bénin (EDSB-V) 2017-2018 [Internet]. Cotonou, Bénin: INSAE/Benin and ICF; 2019. Available from: http://dhsprogram.com/pubs/pdf/FR350/FR350.pdf

11. Instituto Nacional de Estadística. Encuesta de Demografía y Salud (EDSA) [Internet]. 2016. Available from: https://www.ine.gob.bo/index.php/censos-y-banco-de-datos/censos/bases-de-datos-encuestas-sociales/

12. Ministry of Health Burkina Faso, UNICEF, GroundWork. National Iodine Status and Anemia Survey, Burkina Faso 2014. 2014.

13. Ministry of Public Health, ISTEEBU, INSP, UNICEF Burundi, Iodine Global Network. SUSTAINABLE PREVENTION AND CONTROL OF IODINE DEFICIENCY DISORDERS IN BURUNDI: Report of a National IDD Survey in Women of Reproductive Age Conducted in February 2018 [Internet]. 2018. Available from: file:///C:/Users/MisterDee/Dropbox/GAIN_LSFF_meta-analysis/14_Data & analysis/7_Full texts/77_Inventarized reports/BDI_1 2018 COV salt.pdf

14. National Institute of Statistics/Cambodia, Directorate General for Health/Cambodia, ICF International. Cambodia Demographic and Health Survey 2014 [Internet]. Phnom Penh, Cambodia: National Institute of Statistics/Cambodia, Directorate General for Health/Cambodia, and ICF International; 2015. Available from: http://dhsprogram.com/pubs/pdf/FR312/FR312.pdf

15. Ministerio de Saudé Publica. Inquérito sobre os Distúrbios Devidos à Carência em Iodo. 2010.

16. Institut Centrafricain des Statistiques et des Etudes Economiques et Sociales. Central African Republic - Multiple Cluster Indicator Survey 6 [Internet]. 2021. Available from: https://mics-surveys-prod.s3.amazonaws.com/MICS6/West and Central Africa/Central African Republic/2018-2019/Survey findings/French.pdf

17. Institut National de la Statistique des Études Économiques et Démographiques (INSEED), UNICEF. MICS6-Tchad, 2019, Rapport final [Internet]. 2021. Available from: https://mics-surveys-prod.s3.amazonaws.com/MICS6/West%2520and%2520Central%2520Africa/Chad/2019/Survey%25%0A20

18. Li Y, Teng D, Ba J, Chen B, Du J, He L, Lai X, Teng X, Shi X, Li Y, et al. Efficacy and Safety of Long-Term Universal Salt Iodization on Thyroid Disorders: Epidemiological Evidence from 31 Provinces of Mainland China. Thyroid [Internet]. 2020;30:568–79. Available from: file:///C:/Users/MisterDee/Dropbox/GAIN_LSFF_meta-analysis/14_Data & analysis/7_Full texts/77_Inventarized reports/CHN_1 2020 COV salt.pdf

19. Institut National de la Statistique, UNICEF. Enquête par grappes à indicateurs multiples (MICS5 2014-2015), Rapport final [Internet]. Brazzaville, Congo; 2015. Available from: https://mics-surveys-prod.s3.amazonaws.com/MICS5/West and Central Africa/Congo/2014-2015/Final/Congo 2014-15 MICS_French.pdf

20. INS. Enquête par grappes à indicateurs multiples, 2017-2018, rapport de résultats de l’enquête - République Démocratique du Congo [Internet]. 2019. Available from: https://mics-surveys-prod.s3.amazonaws.com/MICS6/West%2520and%2520Central%25%0A20Africa/Congo%252C%2520Democratic%2520Republic%2520of%2520the/2017-2018/Survey%25%0A20findings/Congo%252C%2520Democratic%2520Republic%2520of%2520the%252C%25202017-18%25%0A20MI

21. Institut National de la Statistique, UNICEF. Enquête par grappes à indicateurs multiples - Côte d’Ivoire 2016 [Internet]. 2017. Available from: https://mics-surveys-prod.s3.amazonaws.com/MICS5/West and Central Africa/Côte d%27Ivoire/2016/Final/Cote d%27Ivoire 2016 MICS_French.pdf

22. Instituto Nacional de Investigación en Salud Pública Leopoldo Izquieta Pérez. INFORME PCDDY: RESULTADOS 2016-2017. 2017.

23. Ministry of Health and Population, Global Alliance for Improved Nutrition, UNICEF. Egypt Iodine Survey 2014 / 2015: Summary Report. 2015.

24. Ministerio de Salud Pública y Asistencia Social. Estudio nacional de yoduria, evaluación del estado nutricional y de alimentos fortificados en escolares de primero y segundo grado - El Salvador (2012) [Internet]. 2014. Available from: https://www.salud.gob.sv/archivos/pdf/promocion_salud/material_educativo/componente_nutricion/Estudio_Nac_de_Yoduria_2012_Unidad_Nutrucion.pdf

25. Central Statistical Office, UNICEF. Swaziland Multiple Indicator Cluster Survey 2014. Final Report. [Internet]. 2016. Available from: https://mics-surveys-prod.s3.amazonaws.com/MICS5/Eastern and Southern Africa/Eswatini/2014/Final/Swaziland 2014 MICS Final Report_English.pdf

26. Central Statistical Agency - CSA/Ethiopia, ICF. Ethiopia Demographic and Health Survey 2016 [Internet]. Addis Ababa, Ethiopia: CSA and ICF; 2017. Available from: http://dhsprogram.com/pubs/pdf/FR328/FR328.pdf

27. Ministry of Health Ethiopia, UNICEF, GAIN, World Bank, WFP. Ethiopian National Micronutrient Survey Report, 2016 [Internet]. 2016. Available from: https://www.researchgate.net/publication/316285242_Ethiopian_National_Micro_nutrient_survey

28. Schultz JT, Vatucawaqa PT. Impact of Iron Fortified Flour in Child Bearing Age (CBA) Women in Fiji 2010 report [Internet]. 2012. Available from: https://static1.squarespace.com/static/56424f6ce4b0552eb7fdc4e8/%0At/5759d46e4d088e55539a31e0/1465504911383/Fiji_2010.pdf

29. ICF International. Gabon Enquête Démographique et de Santé 2012 [Internet]. Calverton, Maryland, USA: Direction Générale de la Statistique - DGS/Gabon and ICF International; 2013. Available from: http://dhsprogram.com/pubs/pdf/FR276/FR276.pdf

30. ICF. The Gambia Demographic and Health Survey 2019-20 [Internet]. Banjul, The Gambia: GBoS/ICF; 2021. Available from: https://www.dhsprogram.com/pubs/pdf/FR369/FR369.pdf

31. National Nutrition Agency (NANA), UNICEF, Gambia Bureau of Statistics (GBOS), GroundWork. THE GAMBIA MICRONUTRIENT SURVEY 2018 (GMNS) [Internet]. 2018. Available from: https://groundworkhealth.org/wp-content/uploads/2019/03/GNMS2018-Final-Report_190325.pdf

32. Gerasimov G, Van Der Haar F. Report on the national assessment of iodine nutrition status and iodized salt use in Georgia [Internet]. 2017. Available from: https://www.unicef.org/georgia/media/1246/file/Iodine ENG.pdf

33. Ghana Statistical Service. Multiple Indicator Cluster Survey (MICS2017/18), Survey Findings Report [Internet]. 2018. Available from: https://mics-surveys-prod.s3.amazonaws.com/MICS6/West and Central Africa/Ghana/2017-2018/Survey findings/Ghana 2017-18 MICS Survey Findings Report_English.pdf

34. Ghana Health Services, GAIN, UNICEF. National Iodine Survey Report Ghana 2015 [Internet]. 2017. Available from: https://www.unicef.org/ghana/media/1296/file

35. Instituto de Nutrición de Centro América y Panamá (INCAP). Sistema de Vigilancia Epidemiológica de Salud y Nutrición -SIVESNU- 2016. Guatemala; 2018.

36. ICF. Guinea Demographic and Health Survey (EDS V) 2016-18 [Internet]. Conakry, Guinea: INS/Guinea and ICF; 2019. Available from: http://dhsprogram.com/pubs/pdf/FR353/FR353.pdf

37. Ministério da Economia e Finanças, Direção Geral do Plano/Instituto Nacional de Estatística (INE). Inquérito aos Indicadores Múltiplos (MICS6) 2018-2019, Relatório Final. 2020; Available from: https://mics-surveys-prod.s3.amazonaws.com/MICS6/West and Central Africa/Guinea-Bissau/2018-2019/Survey findings/Guinea Bissau 2018-19 MICS Survey Findings Report_Portuguese.pdf

38. ICF. Haiti Enquête Mortalité, Morbidité et Utilisation des Services 2016-2017 - EMMUS-VI [Internet]. Pétion-Ville/Haïti: IHE/Haiti, ICF; 2018. Available from: http://dhsprogram.com/pubs/pdf/FR326/FR326.pdf

39. Nutrition International, ICCIDD, AIIMS (New Delhi). India Iodine Survey 2018-19 National Report [Internet]. 2018. Available from: https://www.nutritionintl.org/wp-content/uploads/2021/06/India-Iodine-Survey-2018-19-final-report.pdf

40. Delshad H, Mirmiran P, Abdollahi Z, Salehi F, Azizi F. Continuously sustained elimination of iodine deficiency: a quarter of a century success in the Islamic Republic of Iran. J Endocrinol Invest [Internet]. Springer International Publishing; 2018;41:1089–95. Available from: https://doi.org/10.1007/s40618-018-0838-8

41. Ministry of Health, World Health Organization. National Survey to Assess Iodine Deficiency (IDD) Disorders Among Jordanian Children 2010. 2010.

42. Global Alliance for Improved Nutrition, Kazakh Academy of Nutrition. FORTIFICATION ASSESSMENT COVERAGE TOOLKIT (FACT) SURVEY IN KAZAKHSTAN, 2016 [Internet]. 2018. Available from: https://www.gainhealth.org/sites/default/files/publications/documents/fortification-assessement-coveragetoolkit-%0Akazakhstan-2016.pdf

43. Kenya National Bureau of Statistics, Ministry of Health, ICF. Kenya Demographic and Health Survey 2014 [Internet]. Rockville, MD, USA; 2015. Available from: http://dhsprogram.com/pubs/pdf/FR308/FR308.pdf

44. Office KNS. Kiribati Social Development Indicator Survey 2018-19, Survey Findings Report. [Internet]. South Tarawa, Kiribati; 2019. Available from: https://mics-surveys-prod.s3.amazonaws.com/MICS6/East Asia and the Pacific/Kiribati/2018-2019/Survey findings/Kiribati MICS SDIS 2018-19 Survey Findings Report_English.pdf

45. National Statistical Committee of the Kyrgyz Republic, UNICEF. Kyrgyzstan Multiple Indicator Cluster Survey 2018, Survey Findings Report [Internet]. Bishkek, Kyrgyzstan; 2019. Available from: https://mics-surveys-prod.s3.amazonaws.com/MICS6/Europe and Central Asia/Kyrgyzstan/2018/Survey findings/Kyrgyzstan MICS 2018_English.pdf

46. Lao Statistics Bureau, UNICEF, ICF. Lao Social Indicator Survey II 2017, Survey Findings Report [Internet]. Vientiane, Lao PDR: Ministry of Health, Lao Statistics Bureau, ICF; 2018. Available from: http://dhsprogram.com/pubs/pdf/FR356/FR356.pdf

47. ICF. Lesotho Demographic and Health Survey 2014 [Internet]. Maseru, Lesotho: Ministry of Health/Lesotho and ICF International; 2016. Available from: http://dhsprogram.com/pubs/pdf/FR309/FR309.pdf

48. UNICEF, Liberia Institute of Statistics and Geo-Information Services. LIBERIA NATIONAL MICRONUTRIENT SURVEY 2011 [Internet]. 2011. Available from: http://slideplayer.com/slide/9415772/%0A

49. Ministry of Health Macedonia. Iodine and Thyroid Status of Population of Macedonia, 2018. 2018.

50. UNICEF, Office National de Nutrition, Institut Pasteur de Madagascar. Enquête nationale d’iode et du sel à Madagascar (ENISM - 2016).

51. National Statistical Office/Malawi, ICF. Malawi Demographic and Health Survey 2015-16 [Internet]. Zomba, Malawi: National Statistical Office and ICF; 2017. Available from: http://dhsprogram.com/pubs/pdf/FR319/FR319.pdf

52. National Statistical Office, Community Health Services Unit, Centers for Disease control and Prevention, Emory University. Malawi Micronutrient Survey 2015-2016 [Internet]. 2016. Available from: https://www.dhsprogram.com/pubs/pdf/FR319/FR319.m.final.pdf

53. Institut National de la Statistique - INSTAT, Cellule de Planification et de la Statistique - Secteur Santé-Développement, ICF. Mali Demographic and Health Survey 2018 [Internet]. Bamako, Mali: INSTAT/CPS/SS-DS-PF and ICF; 2019. Available from: http://dhsprogram.com/pubs/pdf/FR358/FR358.pdf

54. Ministère de l’Économie et des Finances, Office National de la Statistique. Enquête par grappes à indicateurs multiples MICS5 2015 [Internet]. 2017. Available from: https://mics-surveys-prod.s3.amazonaws.com/MICS5/West and Central Africa/Mauritania/2015/Final/Mauritania 2015 MICS_French.pdf

55. National Centre of Public Health of the Ministry of Health of the Republic of Moldova, UNICEF. 2012 Republic of Moldova Multiple Indicator Cluster Survey, Final Report [Internet]. Chișinău, Republic of Moldova; 2014. Available from: https://mics-surveys-prod.s3.amazonaws.com/MICS4/Europe and Central Asia/Moldova%2C Republic of/2012/Final/Moldova 2012 MICS_English.pdf

56. National Statistics Office. Social Indicator Sample Survey-2018, Survey Findings Report [Internet]. Ulaanbaatar, Mongolia; 2019. Available from: https://mics-surveys-prod.s3.amazonaws.com/MICS6/East Asia and the Pacific/Mongolia/2018/Survey findings/SISS2018-MICS6 SFR_English.pdf

57. Programme National de Nutrition. Enquête Nationale sur la Nutrition, Diversité alimentaire, Carence en Fer, Carence en Vitamine A, Carence en Iode - Maroc 2019. 2020.

58. Institut Nacional de Estádistica, ICF. Moçambique Inquérito Demográfico e de Saúde 2011 [Internet]. Calverton, Maryland, USA: MISA/Moçambique, INE/Moçambique and ICF International; 2013. Available from: http://dhsprogram.com/pubs/pdf/FR266/FR266.pdf

59. National Nutrition Centre, Ministry of Health. Myanmar Micronutrient and Food Consumption Survey 2017-18 [Internet]. Available from: https://www.mohs.gov.mm/page/7339

60. Ministry of Health and Social Services, ICF. Namibia Demographic and Health Survey 2013 [Internet]. Windhoek, Namibia: MoHSS/Namibia and ICF International; 2014. Available from: http://dhsprogram.com/pubs/pdf/FR298/FR298.pdf

61. Ministry of Health, New ERA, ICF. Nepal Demographic and Health Survey 2016 [Internet]. Kathmandu, Nepal: MOH/Nepal, New ERA, and ICF; 2017. Available from: http://dhsprogram.com/pubs/pdf/FR336/FR336.pdf

62. UNICEF, World Food Programme, Institut National de la Statistique. Rapport final de l’Evaluation nationale de la situation nutritionnelle par la méthodologie SMART - Niger 2018 [Internet]. 2019. Available from: https://www.humanitarianresponse.info/sites/www.humanitarianresponse.info/files/documents/files/rapport_enquete_smart_2018_vf.pdf

63. ICF. Nigeria Demographic and Health Survey 2018 - Final Report [Internet]. Abuja, Nigeria: NPC and ICF; 2019. Available from: http://dhsprogram.com/pubs/pdf/FR359/FR359.pdf

64. National Centre for Statistics and Information. Multiple Indicator Cluster Survey 2014, Key Findings, Muscat, Oman [Internet]. 2015. Available from: https://mics-surveys-prod.s3.amazonaws.com/MICS5/Middle East and North Africa/Oman/2014/Key findings/Oman 2014 MICS KFR_English.pdf

65. Statistics PCB of. Palestinian Multiple Indicator Cluster Survey 2019-2020, Survey Findings Report [Internet]. Ramallah, Palestine; 2021. Available from: https://mics-surveys-prod.s3.amazonaws.com/MICS6/Middle East and North Africa/State of Palestine/2019-2020/Survey findings/State of Palestine 2019-20 Survey Findings Report_English.pdf

66. National Statistical Office, ICF. Papua New Guinea Demographic and Health Survey 2016-18 [Internet]. Port Moresby, Papua New Guinea: NSO and ICF; 2019. Available from: https://www.dhsprogram.com/pubs/pdf/FR364/FR364.pdf

67. Instituto Nacional de Alimentación y Nutrición. Niveles de Yodo en muestras de sal en hogares (Encuesta a escolares) en las Regiones Sanitarias en Paraguay: 2008-19 [Internet]. 2020. Available from: https://www.inan.gov.py/site/?page_id=501

68. Instituto Nacional de Estadística e Informática - INEI. Encuesta Nacional Demografía y Salud Familiar (ENDES) 2019 [Internet]. 2019. Available from: https://www.inei.gob.pe/media/MenuRecursivo/publicaciones_digitales/Est/Endes2019/

69. Department of Science and Technology - Food and Nutrition Research Institute (DOST-FNRI). 2018 Expanded National Nutrition Survey [Internet]. 2020. Available from: http://enutrition.fnri.dost.gov.ph/site/uploads/2018 Expanded National Nutrition Survey-SAMAR.pdf

70. World Health Organization, UNICEF, ICCIDD. Assessment of Iodine Deficiency Disorders and Monitoring their Elimination in Qatar [Internet]. 2014. Available from: file:///C:/Users/MisterDee/Dropbox/GAIN_LSFF_meta-analysis/14_Data & analysis/7_Full texts/77_Inventarized reports/QAT_1 2014 COV salt.pdf

71. National Institute of Statistics of Rwanda, Ministry of Finance and Economic Planning, Ministry of Health, ICF. Rwanda Demographic and Health Survey 2014-15 [Internet]. Kigali, Rwanda: National Institute of Statistics of Rwanda, Ministry of Finance and Economic Planning/Rwanda, Ministry of Health/Rwanda, and ICF International; 2016. Available from: http://dhsprogram.com/pubs/pdf/FR316/FR316.pdf

72. Instituto Nacional de Estatística, UNICEF. Inquérito de Indicadores Múltiplos 2019, Relatório final - São Tomé e Príncipe. 2020; Available from: https://mics-surveys-prod.s3.amazonaws.com/MICS6/West and Central Africa/Sao Tome and Principe/2019/Survey findings/Sao Tome e Principe 2019 MICS Survey Findings Report_Portuguese.pdf

73. Al-Shangiti A, Al-Shehri S, Haridi H, Hussein I, Al-Dakheel M, Al-Bashir B, Gassem M. Prevalence of iodine deficiency disoriders among school children in Saudi Arabia: results of a national iodine nutrition study. East Mediterr Heal J. 2017;22:301–8.

74. Agence Nationale de la Statistique et de la Démographie (ANSD) [Sénégal], ICF. Senegal: Enquête Démographique et de Santé Continue (EDS- Continue) 2019 [Internet]. Dakar/ Sénégal: ANSD/ICF; 2020. Available from: https://www.dhsprogram.com/pubs/pdf/FR368/FR368.pdf

75. ICF. Sierra Leone Demographic and Health Survey 2019 [Internet]. Freetown/Sierra Leone: StatsSL/ICF; 2020. Available from: https://www.dhsprogram.com/pubs/pdf/FR365/FR365.pdf

76. Ministry of Health and Medical Services, Statistics Office. Solomon Islands Demographic and Health Survey 2015 [Internet]. 2017. Available from: https://purl.org/spc/digilib/doc/zmgy8

77. Ministry of Health (FGS/FMS/Somaliand), UNICEF, Brandpro, GroundWork. Somali Micronutrient Survey 2019 [Internet]. 2019. Available from: https://www.unicef.org/somalia/reports/somalia-micronutrient-survey-2019

78. National Department of Health, Statistics South Africa, South African Medical Research Council, ICF. South Africa Demographic and Health Survey 2016 [Internet]. Pretoria: National Department of Health - NDoH - ICF; 2019. Available from: http://dhsprogram.com/pubs/pdf/FR337/FR337.pdf

79. Department of Census and Statistics, Ministry of Health Nutrition and Indigenous Medicine. Sri Lanka Demographic and Health Survey 2016 [Internet]. 2017. Available from: http://www.statistics.gov.lk/Resource/en/Health/DemographicAndHealthSurveyReport-2016-Contents.pdf

80. Central Bureau of Statistics, UNICEF. Multiple Indicator Cluster Survey 2014 of Sudan, Final Report [Internet]. Khartoum, Sudan; 2016. Available from: https://mics-surveys-prod.s3.amazonaws.com/MICS5/Middle East and North Africa/Sudan/2014/Final/Sudan 2014 MICS_English.pdf

81. Statistical Agency under the President of the Republic of Tajikistan, Ministry of Health and Social Protection of Population of the Republic of Tajikistan, ICF. Tajikistan-Demographic and Health Survey 2017 [Internet]. 2018. Available from: https://www.dhsprogram.com/pubs/pdf/FR341/FR341.pdf

82. Ministry of Health Gender Elderly and Children, Office of the Chief Government Statistician Ministry of Health (MoH), Tanzania Food and Nutrition Centre, National Bureau of Statistics, UNICEF. Tanzania National Nutrition Survey using SMART Methodology (TNNS) 2018 [Internet]. 2019. Available from: https://www.unicef.org/tanzania/reports/tanzania-national-nutrition-survey-2018

83. National Statistical Office of Thailand. Thailand Multiple Indicator Cluster Survey 2019, Survey Findings Report [Internet]. Bangkok, Thailand; 2020. Available from: https://mics-surveys-prod.s3.amazonaws.com/MICS6/East Asia and the Pacific/Thailand/2019/Survey findings/Thailand 2019 MICS Survey Findings Report_English.pdf

84. Institut National de la Statistique et des Etudes Economiques et Démographiques, (INSEED). MICS6 TOGO, 2017, Rapport final [Internet]. Lomé, Togo; 2018. Available from: https://mics-surveys-prod.s3.amazonaws.com/MICS6/West and Central Africa/Togo/2017/Survey findings/Togo 2017 MICS SFR-v2_French.pdf

85. El Ati J, Doggui R, Nabli M, Lahmar L, Fourati A, Medimagh AA, Treissac P. Enquête nationale sur la prévalence des troubles liés à la carence en iode chez les enfants scolarisés âgés de 6 à 12 ans en Tunisie 2012. 2015.

86. The State Committee of Statistics of Turkmenistan, UNICEF. Turkmenistan Multiple Indicator Cluster Survey 2015-16, Final Report [Internet]. 2017 [cited 2019 Dec 18]. Available from: https://mics-surveys-prod.s3.amazonaws.com/MICS5/Europe and Central Asia/Turkmenistan/2015-2016/Final/Turkmenistan 2015-2016 MICS_English.pdf

87. Uganda Bureau of Statistics, ICF. Uganda Demographic and Health Survey 2016 [Internet]. 2018. Available from: https://www.dhsprogram.com/pubs/pdf/FR333/FR333.pdf

88. Ministry of Health Uzbekistan, UNICEF, GroundWork. Uzbekistan Nutrition Survey 2017 [Internet]. 2019. Available from: https://groundworkhealth.org/wp-content/uploads/2019/11/Uzbekistan_Nutrition_Survey_en_web.pdf

89. General Statistical Office (GSO). Viet Nam Multiple Indicator Cluster Survey 2011, Final Report [Internet]. Ha Noi, Vietnam; 2011. Available from: https://mics-surveys-prod.s3.amazonaws.com/MICS4/East Asia and the Pacific/Viet Nam/2010-2011/Final/Viet Nam 2011 MICS_English.pdf

90. Ministry of Public Health and Population, UNICEF, USAID, EU, GAIN. Yemen National Iodine Deficiency Survey 2015. 2015.

91. Central Statistical Office, Ministry of Health, ICF. Zambia Demographic and Health Survey 2013-14 [Internet]. Rockville, Maryland, USA: Central Statistical Office/Zambia, Ministry of Health/Zambia, and ICF International; 2015. Available from: http://dhsprogram.com/pubs/pdf/FR304/FR304.pdf

92. Zimbabwe National Statistics Agency, UNICEF. Zimbabwe Multiple Indicator Cluster Survey 2019, Survey Findings Report [Internet]. Harare, Zimbabwe; 2019. Available from: https://mics-surveys-prod.s3.amazonaws.com/MICS6/Eastern and Southern Africa/Zimbabwe/2019/Survey findings/Zimbabwe 2019 MICS Survey Findings Report-31012020_English.pdf

93. University of Ghana, GroundWork, University of Wisconsin-Madison, KEMRI-Wellcome, UNICEF. Ghana Micronutrient Survey GMS 2017 [Internet]. Open Science Framework; 2017 [cited 2018 Sep 7]. Available from: https://groundworkhealth.org/wp-content/uploads/2018/06/UoG-GroundWork_2017-GHANA-MICRONUTRIENT-SURVEY_Final_180607.pdf

94. Jordan Ministry of Health, Global Alliance for Improved Nutrition, United States Centers of Disease Control and Prevention, UNICEF. National Micronutrient Survey , Jordan 2010 [Internet]. 2010. Available from: https://www.gainhealth.org/sites/default/files/publications/documents/national-micronutrient-survey-jordan-2010.pdf

95. Ministry of Health, National Center for Public Health, UNICEF. Nutrition Status of the Population of Mongolia: Fifth National Nutrition Survey Report, 2017 [Internet]. 2017. Available from: https://www.unicef.org/mongolia/media/1116/file/NNS_V_undsen_tailan_EN.pdf

96. Ministry of Health (Mozambique), Helen Keller International, Global Alliance for Improved Nutrition. Mozambique Micronutrient Survey 2012-13 [Internet]. 2013. Available from: https://groundworkhealth.org/wp-content/uploads/2021/04/Mozambique-Micronutrient-2012-Survey-Report_170705_FINAL.pdf

97. Ministry of Health and Population Nepal, New ERA, UNICEF, European Union, USAID, CDC. Nepal National Micronutrient Status Survey 2016 [Internet]. 2018. Available from: https://www.unicef.org/nepal/media/1206/file/Nepal%2520National%2520Micronutrient%2520Status%2520Survey%25%0A20Report%25202016.pdf

98. Global Alliance for Improved Nutrition, Cellule de Lutte contre la Malnutrition. Couverture nationale de la farine de blé et de l’huile chez les femmes en âge de procréer au Sénégal: compte rendu sur l’outil d’évaluation de la couverture des aliments enrichis (FACT) [Internet]. 2014. Available from: https://www.gainhealth.org/resources/datasets/fortification-assessment-coverage-toolkit-fact-survey-senegal

99. Ministry of Health and Sanitation (Sierra Leone), Helen Keller International, UNICEF, WHO. 2013 Sierra Leone Micronutrient Survey [Internet]. Freetown, Sierra Leone 2015; 2015. Available from: http://groundworkhealth.org/wp-content/uploads/2016/03/SLMS-Report_FINAL_151203.pdf

100. Imhoff-Kunsch B, Shakya I, Namohunu SAD, Pitaboe A, Wong P, Tsang BL, Codling K, Foley J, Pachón H. Potential dietary contributions from rice and wheat flour fortification in the Solomon Islands: results from the 2012-2013 Household Income and Expenditure Survey. Food Nutr Bull. SAGE Publications Sage CA: Los Angeles, CA; 2019;40:71–86.

101. Africa Academy of Public Health, US Centers of Disease Control and Prevention, Global Allliance for Improved Nutrition. National Fortification Assessment Coverage Tool (FACT) Survey in Tanzania, 2015 [Internet]. 2015. Available from: https://www.gainhealth.org/resources/reports-and-publications/fortification-assessment-coverage-toolkit-fact-survey-tanzania

102. Ministry of Health and Child Care, UNICEF. Smart Survey 2019 Results of The Emergency Nutrition Assessment in 11 Selected Districts, Zimbabwe March 2019 [Internet]. 2019. Available from: https://www.humanitarianresponse.info/sites/www.humanitarianresponse.info/files/2019/04/SMART-REPORT-2019-Final_1.pdf

103. Fiedler JL, Lividini K, Bermudez OI. Estimating the impact of vitamin A-fortified vegetable oil in Bangladesh in the absence of dietary assessment data. Public Health Nutr. Cambridge University Press; 2015;18:414–20.

104. Global Allliance for Improved Nutrition, Oxford Policy Management. REPORT FORTIFICATION ASSESSMENT COVERAGE TOOLKIT ( FACT ) SURVEY IN PAKISTAN [Internet]. 2018. Available from: https://www.gainhealth.org/sites/default/files/publications/documents/pakistan-fact-survey-2017-final-report-aug2018-corrected.pdf

105. Aaron GJ, Friesen VM, Jungjohann S, Garrett GS, Neufeld LM, Myatt M. Coverage of Large-Scale Food Fortification of Edible Oil, Wheat Flour, and Maize Flour Varies Greatly by Vehicle and Country but Is Consistently Lower among the Most Vulnerable: Results from Coverage Surveys in 8 Countries. J Nutr. 2017;147:984S-994S.

106. US Centers of Disease Control and Prevention, Global Alliance for Improved Nutrition, Makerere University. Fortification Assessment Coverage Tool ( FACT ) Survey in Uganda , 2015 [Internet]. 2017. Available from: https://www.gainhealth.org/sites/default/files/publications/documents/fortification-assessement-coverage-toolkit-uganda-2015.pdf

107. Instituto de Nutrición de Centro América y Panamá (INCAP). Informe del Sistema de Vigilancia Epidemiológica de Salud y Nutrición -SIVESNU- agosto 2018 – mayo 2019 – Módulo 1: Introducción y datos del hogar, Informe final. 2019.
