# Supplementary material for: Global Coverage of Mandatory Large-Scale Food Fortification Programs: A Systematic Review and Meta-Analysis
Source: Adv Nutr. 2023 Jul 25;14(5):1197–210. doi: 10.1016/j.advnut.2023.07.004 (PMC10509437; doi:10.1016/j.advnut.2023.07.004)
Supplement: Multimedia component4 [file mmc4.pdf]

Forest plots presenting meta-analysis results for mandatory fortification programs; (A) proportion of households consuming fortified food vehicle, (B) proportion of households consuming fortifiable food vehicle, (C) proportion of households consuming fortified food vehicle, and (D) proportion of households consuming adequately fortified food vehicle

Salt

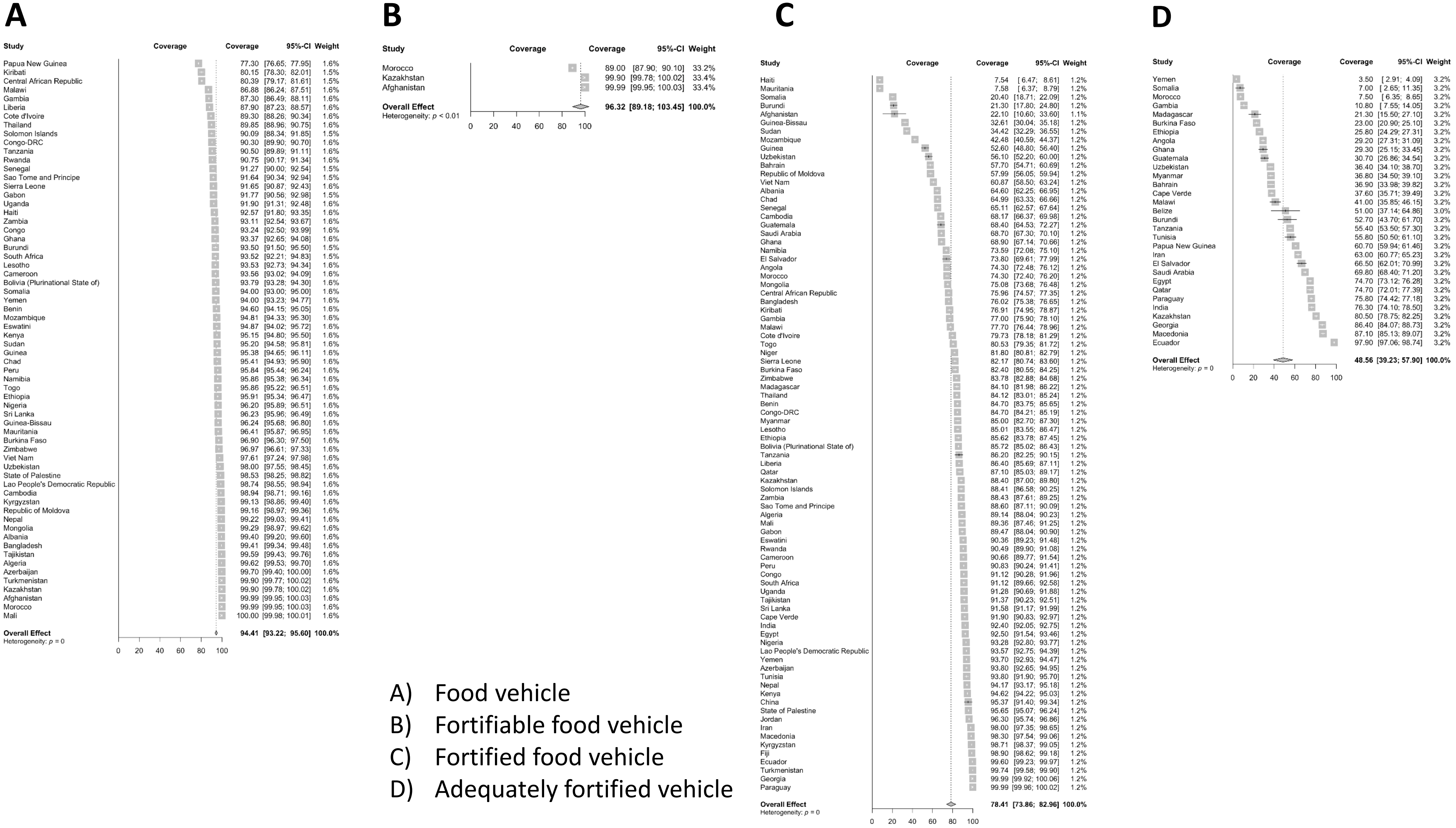

A) Food vehicle
B) Fortifiable food vehicle
C) Fortified food vehicle
D) Adequately fortified vehicle

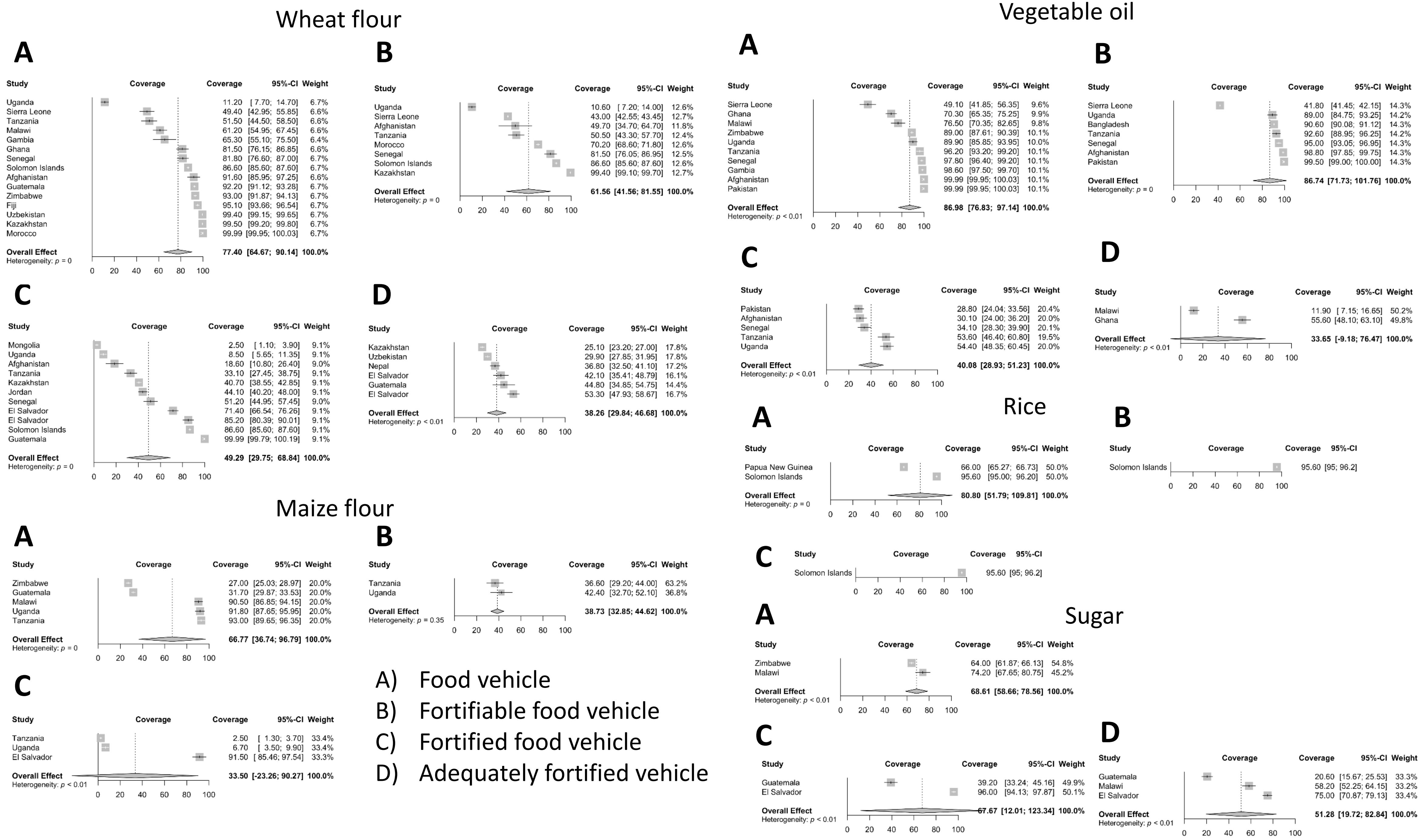
Wheat flour
A
Study Coverage Coverage 95%-CI Weight
Uganda 11.20 [7.70; 14.70] 6.7%
Sierra Leone 49.40 [42.95; 55.85] 6.6%
Tanzania 51.50 [44.50; 58.50] 6.6%
Malawi 61.20 [54.95; 67.45] 6.6%
Gambia 65.30 [55.10; 75.50] 6.4%
Ghana 81.50 [76.15; 86.85] 6.6%
Senegal 81.80 [76.60; 87.00] 6.7%
Solomon Islands 86.60 [85.60; 87.60] 6.7%
Afghanistan 91.60 [85.95; 97.25] 6.6%
Guatemala 92.20 [91.12; 93.28] 6.7%
Zimbabwe 93.00 [91.87; 94.13] 6.7%
Fiji 95.10 [93.66; 96.54] 6.7%
Uzbekistan 99.40 [99.15; 99.65] 6.7%
Kazakhstan 99.50 [99.20; 99.80] 6.7%
Morocco 99.99 [99.95; 100.03] 6.7%
Overall Effect 77.40 [64.67; 90.14] 100.0%
Heterogeneity: p = 0
0 20 40 60 80 100
B
Study Coverage Coverage 95%-CI Weight
Uganda 10.60 [7.20; 14.00] 12.6%
Sierra Leone 43.00 [42.55; 43.45] 12.7%
Afghanistan 49.70 [34.70; 64.70] 11.8%
Tanzania 50.50 [43.30; 57.70] 12.4%
Morocco 70.20 [68.60; 71.80] 12.6%
Senegal 81.50 [76.05; 86.95] 12.5%
Solomon Islands 86.60 [85.60; 87.60] 12.6%
Kazakhstan 99.40 [99.10; 99.70] 12.7%
Overall Effect 61.56 [41.56; 81.55] 100.0%
Heterogeneity: p = 0
0 20 40 60 80 100
C
Study Coverage Coverage 95%-CI Weight
Mongolia 2.50 [1.10; 3.90] 9.1%
Uganda 8.50 [5.65; 11.35] 9.1%
Afghanistan 18.60 [10.80; 26.40] 9.0%
Tanzania 33.10 [27.45; 38.75] 9.1%
Kazakhstan 40.70 [38.55; 42.85] 9.1%
Jordan 44.10 [40.20; 48.00] 9.1%
Senegal 51.20 [44.95; 57.45] 9.0%
El Salvador 71.40 [66.54; 76.26] 9.1%
El Salvador 85.20 [80.39; 90.01] 9.1%
Solomon Islands 86.60 [85.60; 87.60] 9.1%
Guatemala 99.99 [99.79; 100.19] 9.1%
Overall Effect 49.29 [29.75; 68.84] 100.0%
Heterogeneity: p = 0
0 20 40 60 80 100
D
Study Coverage Coverage 95%-CI Weight
Kazakhstan 25.10 [23.20; 27.00] 17.8%
Uzbekistan 29.90 [27.85; 31.95] 17.8%
Nepal 36.80 [32.50; 41.10] 17.2%
El Salvador 42.10 [35.41; 48.79] 16.1%
Guatemala 44.80 [34.85; 54.75] 14.4%
El Salvador 53.30 [47.93; 58.67] 16.7%
Overall Effect 38.26 [29.84; 46.68] 100.0%
Heterogeneity: p < 0.01
0 20 40 60 80 100
Maize flour
A
Study Coverage Coverage 95%-CI Weight
Zimbabwe 27.00 [25.03; 28.97] 20.0%
Guatemala 31.70 [29.87; 33.53] 20.0%
Malawi 90.50 [86.85; 94.15] 20.0%
Uganda 91.80 [87.65; 95.95] 20.0%
Tanzania 93.00 [89.65; 96.35] 20.0%
Overall Effect 66.77 [36.74; 96.79] 100.0%
Heterogeneity: p = 0
0 20 40 60 80 100
B
Study Coverage Coverage 95%-CI Weight
Tanzania 36.60 [29.20; 44.00] 63.2%
Uganda 42.40 [32.70; 52.10] 36.8%
Overall Effect 38.73 [32.85; 44.62] 100.0%
Heterogeneity: p = 0.35
0 20 40 60 80 100
C
Study Coverage Coverage 95%-CI Weight
Tanzania 2.50 [1.30; 3.70] 33.4%
Uganda 6.70 [3.50; 9.90] 33.4%
El Salvador 91.50 [85.46; 97.54] 33.3%
Overall Effect 33.50 [-23.26; 90.27] 100.0%
Heterogeneity: p < 0.01
0 20 40 60 80 100
A) Food vehicle
B) Fortifiable food vehicle
C) Fortified food vehicle
D) Adequately fortified vehicle
Vegetable oil
A
Study Coverage Coverage 95%-CI Weight
Sierra Leone 49.10 [41.85; 56.35] 9.6%
Ghana 70.30 [65.35; 75.25] 9.9%
Malawi 76.50 [70.35; 82.65] 9.8%
Zimbabwe 89.00 [87.61; 90.39] 10.1%
Uganda 89.90 [85.85; 93.95] 10.0%
Tanzania 96.20 [93.20; 99.20] 10.1%
Senegal 97.80 [96.40; 99.20] 10.1%
Gambia 98.60 [97.50; 99.70] 10.1%
Afghanistan 99.99 [99.95; 100.03] 10.1%
Pakistan 99.99 [99.95; 100.03] 10.1%
Overall Effect 86.98 [76.83; 97.14] 100.0%
Heterogeneity: p < 0.01
0 20 40 60 80 100
B
Study Coverage Coverage 95%-CI Weight
Sierra Leone 41.80 [41.45; 42.15] 14.3%
Uganda 89.00 [84.75; 93.25] 14.2%
Bangladesh 90.60 [90.08; 91.12] 14.3%
Tanzania 92.60 [88.95; 96.25] 14.2%
Senegal 95.00 [93.05; 96.95] 14.3%
Afghanistan 98.80 [97.85; 99.75] 14.3%
Pakistan 99.50 [99.00; 100.00] 14.3%
Overall Effect 86.74 [71.73; 101.76] 100.0%
Heterogeneity: p = 0
0 20 40 60 80 100
C
Study Coverage Coverage 95%-CI Weight
Pakistan 28.80 [24.04; 33.56] 20.4%
Afghanistan 30.10 [24.00; 36.20] 20.0%
Senegal 34.10 [28.30; 39.90] 20.1%
Tanzania 53.60 [46.40; 60.80] 19.5%
Uganda 54.40 [48.35; 60.45] 20.0%
Overall Effect 40.08 [28.93; 51.23] 100.0%
Heterogeneity: p < 0.01
0 20 40 60 80 100
D
Study Coverage Coverage 95%-CI Weight
Malawi 11.90 [7.15; 16.65] 50.2%
Ghana 55.60 [48.10; 63.10] 49.8%
Overall Effect 33.65 [-9.18; 76.47] 100.0%
Heterogeneity: p < 0.01
0 20 40 60 80 100
Rice
A
Study Coverage Coverage 95%-CI Weight
Papua New Guinea 66.00 [65.27; 66.73] 50.0%
Solomon Islands 95.60 [95.00; 96.20] 50.0%
Overall Effect 80.80 [51.79; 109.81] 100.0%
Heterogeneity: p = 0
0 20 40 60 80 100
B
Study Coverage Coverage 95%-CI
Solomon Islands 95.60 [95; 96.2]
0 20 40 60 80 100
C
Study Coverage Coverage 95%-CI
Solomon Islands 95.60 [95; 96.2]
0 20 40 60 80 100
Sugar
A
Study Coverage Coverage 95%-CI Weight
Zimbabwe 64.00 [61.87; 66.13] 54.8%
Malawi 74.20 [67.65; 80.75] 45.2%
Overall Effect 68.61 [58.66; 78.56] 100.0%
Heterogeneity: p < 0.01
0 20 40 60 80 100
C
Study Coverage Coverage 95%-CI Weight
Guatemala 39.20 [33.24; 45.16] 49.9%
El Salvador 96.00 [94.13; 97.87] 50.1%
Overall Effect 67.67 [12.01; 123.34] 100.0%
Heterogeneity: p < 0.01
0 20 40 60 80 100
D
Study Coverage Coverage 95%-CI Weight
Guatemala 20.60 [15.67; 25.53] 33.3%
Malawi 58.20 [52.25; 64.15] 33.2%
El Salvador 75.00 [70.87; 79.13] 33.4%
Overall Effect 51.28 [19.72; 82.84] 100.0%
Heterogeneity: p < 0.01
0 20 40 60 80 100
